# Mapping cross-variant neutralizing sites on the SARS-CoV-2 spike protein

**DOI:** 10.1080/22221751.2021.2024455

**Published:** 2022-01-24

**Authors:** Shiqi Xu, Yifan Wang, Yanxing Wang, Chao Zhang, Qin Hong, Chenjian Gu, Rong Xu, Tingfeng Wang, Yong Yang, Jinkai Zang, Yu Zhou, Zuyang Li, Qixing Liu, Bingjie Zhou, Lulu Bai, Yuanfei Zhu, Qiang Deng, Haikun Wang, Dimitri Lavillette, Gary Wong, Youhua Xie, Yao Cong, Zhong Huang

**Affiliations:** a CAS Key Laboratory of Molecular Virology & Immunology, Institut Pasteur of Shanghai, Chinese Academy of Sciences, University of Chinese Academy of Sciences, Shanghai, People’s Republic of China; b State Key Laboratory of Molecular Biology, National Center for Protein Science Shanghai, Shanghai Institute of Biochemistry and Cell Biology, Center for Excellence in Molecular Cell Science, Chinese Academy of Sciences, University of Chinese Academy of Sciences, Shanghai, People’s Republic of China; c Key Laboratory of Medical Molecular Virology (MOE/NHC/CAMS), Department of Medical Microbiology and Parasitology, School of Basic Medical Sciences, Shanghai Medical College, Fudan University, Shanghai, People’s Republic of China; d BSL-3 Laboratory of Fudan University, School of Basic Medical Sciences, Shanghai Medical College, Fudan University, Shanghai, People’s Republic of China

**Keywords:** SARS-CoV-2, antibody, SD1, epitope, Cryo-EM

## Abstract

The emergence of multiple severe acute respiratory syndrome coronavirus 2 (SARS-CoV-2) variants of concern threatens the efficacy of currently approved vaccines and authorized therapeutic monoclonal antibodies (MAbs). It is hence important to continue searching for SARS-CoV-2 broadly neutralizing MAbs and defining their epitopes. Here, we isolate 9 neutralizing mouse MAbs raised against the spike protein of a SARS-CoV-2 prototype strain and evaluate their neutralizing potency towards a panel of variants, including B.1.1.7, B.1.351, B.1.617.1, and B.1.617.2. By using a combination of biochemical, virological, and cryo-EM structural analyses, we identify three types of cross-variant neutralizing MAbs, represented by S5D2, S5G2, and S3H3, respectively, and further define their epitopes. S5D2 binds the top lateral edge of the receptor-binding motif within the receptor-binding domain (RBD) with a binding footprint centred around the loop^477–489^, and efficiently neutralizes all variant pseudoviruses, but the potency against B.1.617.2 was observed to decrease significantly. S5G2 targets the highly conserved RBD core region and exhibits comparable neutralization towards the variant panel. S3H3 binds a previously unreported epitope located within the evolutionarily stable SD1 region and is able to near equally neutralize all of the variants tested. Our work thus defines three distinct cross-variant neutralizing sites on the SARS-CoV-2 spike protein, providing guidance for design and development of broadly effective vaccines and MAb-based therapies.

## Introduction

The unprecedented pandemic of coronavirus disease 2019 (COVID-19), caused by severe acute respiratory syndrome coronavirus 2 (SARS-CoV-2) [1–3], has led to a huge number of infections and death worldwide as well as enormous social and economic disruption [3]. SARS-CoV-2 belongs to the *Betacoronavirus* genus within the *Coronaviridae* family [4]. Like other coronaviruses, SARS-CoV-2 possesses a single-stranded, positive-sense RNA genome and an outer envelope made of membrane (M), envelope (E), and spike (S) proteins. The S protein is a single-span transmembrane protein that protrudes from the surface of SARS-CoV-2 virions to engage the host receptor, human angiotensin-converting enzyme 2 (ACE2) [1,5–8]. The ectodomain of the S protein consists of a receptor-binding subunit termed S1 and a membrane-fusion subunit termed S2. The S1 subunit can be divided into four distinct domains, including the N-terminal domain (NTD), the receptor-binding domain (RBD), the subdomain 1 (SD1) and the subdomain 2 (SD2). RBD can be further divided into a core domain and a receptor-binding motif (RBM) that directly interacts with the ACE2 receptor [1,5–8]. The S protein of SARS-CoV-2 forms homotrimers on the virion surface and likely exists in two major states, namely the “closed” state with three RBD down (receptor-inaccessible) and the “open” state with one RBD up (receptor-accessible) [9–14].

Monoclonal antibodies (MAbs) that can neutralize SARS-CoV-2 infection in vitro represent a viable option for anti-SARS-CoV-2 drug development. Until now, a large number of SARS-CoV-2 neutralizing MAbs have been identified and tested in preclinical studies, and some of them have advanced into clinical trials [15]. Notably, six SARS-CoV-2 MAbs has recently received an Emergency Use Authorization (EUA) by the United States or South Korea for early therapy of COVID19 [15]. Almost all of SARS-CoV-2 neutralizing MAbs identified thus far target either the RBD or the NTD regions [15–20]. In particular, all MAbs authorized or in clinical trials are directed to the SARS-CoV-2 RBD [15,18,21,22]. RBD-directed neutralizing MAbs targets multiple overlapping and non-overlapping epitopes and can be classified into three categories based on their ACE2-blocking capacity and RBD conformation preference, including class 1 that blocks ACE2 and binds only to “up” RBDs, class 2 that also blocks ACE2 but binds both “up” and “down” RBDs, and class 3 that recognizes both “up” and “down” RBDs but does not interfere with ACE2 binding [16–19]. In general, RBD-targeting MAb’s neutralization potency correlates with its ACE2-blocking efficiency [21]. For NTD-directed potent neutralizing MAbs, their binding epitopes appear to highly overlap, forming an antigenic supersite [19,23]. Besides RBD- and NTD-directed MAbs, neutralizing MAbs that bind the S2 region have also been reported, however, the neutralizing potency of these S2-directed MAbs is very low [24–26].

SARS-CoV-2 has evolved considerably since its first identification in late 2019. A number of SARS-CoV-2 variants of interest (VOI) and variants of concern (VOC) have emerged, such as B.1.1.7 lineage that arose in the United Kingdom, B.1.1.28 lineage (also called “P.1”) in Brazil, and B.1.351 lineage in South Africa [20,27]. These variants carry multiple mutations in the RBD and NTD regions of the S protein, leading to their increased resistance to neutralization by MAbs raised against the original strain in the early phase of the pandemic [20,27]. In particular, the B.1.351 variant has been found to be refractory to some MAbs approved or in development [28–32]. Specifically, the antibody Bamlanivimab of Lilly failed to neutralize B.1.351 [30,31], and as a result, the EUA of Bamlanivimab monotherapy was recently revoked by the FDA. The B.1.617 lineage, emerged recently in India [33], includes three main subtypes, namely B.1.617.1, B.1.617.2 and B.1.617.3 [20,27]. The B.1.617.2 variant (recently renamed “Delta”) has spread rapidly and is now the predominant circulating strain worldwide [34–36]. The B.1.617.2 variant carries a new mutation, T478K, in its RBD, but the impact of this mutation or the variant as a whole on MAbs’ neutralization potency has not been adequately examined. Nonetheless, as SARS-CoV-2 evolves constantly, it is important to continue searching for broadly neutralizing MAbs and defining their epitopes, in order to develop broad-spectrum antiviral therapies and to guide the design of broadly effective anti-SARS-CoV-2 vaccines.

In this study, we isolated 9 neutralizing MAbs from mice immunized with the S protein of an original SARS-CoV-2 strain and subsequently evaluated their neutralization potency against a panel of variants including B.1.1.7, B.1.351, B.1.617.1, and B.1.617.2. Three types of cross-variant neutralizing MAbs were identified: (1) RBM-binding MAb that blocks ACE2 and neutralizes potently the panel of variants except B.1.617.2; (2) RBD core-targeting MAb that blocks ACE2 and neutralizes all of variants tested; and (3) non-RBD-, non-NTD-reactive MAb that cross-neutralizes all variants without ACE2 blockade. We further performed cryo-electron microscopy (cryo-EM) structural study to define the binding epitopes of antibodies S5D2 and S3H3, representing the type 1 and 3 broadly neutralizing MAbs, respectively. Cryo-EM structures of B.1.351 S trimer in complex with the Fab of S5D2 were resolved up to 3.3 Å, revealing not only the binding epitope for S5D2 but also the potential mechanism of immune escape by B.1.617.2. Significantly, cryo-EM reconstruction of the Fab of S3H3 revealed that the antibody bound in the evolutionally stable SD1 region of the B.1.351 S trimer, thus defining a previously unreported broadly neutralizing epitope. These SARS-CoV-2 cross-variant neutralizing MAbs and their epitope information will have important implications for development of broadly effective vaccines and antiviral therapies.

## Materials and methods

### Cells and viruses

SP2/0 (mouse myeloma cell line) was cultured in RPMI 1640 medium (Gibco, Thermo Fisher Scientific, USA) supplemented with 10% fetal bovine serum (FBS; Gibco). VeroE6 (African green monkey kidney cell line) was cultured in DMEM (Gibco) with 10% FBS. HEK 293T (human embryonic kidney cell line) over-expressing human ACE2 (293T-hACE2) and VeroE6 over-expressing hACE2 (VeroE6-hACE2) cell lines were constructed in a previous study [37]. HEK 293F suspension cells (Gibco) were grown in FreeStyle 293 expression medium (Gibco). SARS-CoV-2 strain nCoV-SH01 (GenBank number: MT121215.1) [38] was propagated in VeroE6 cells and viral titre was expressed as plaque forming units (pfu) per mL.

### Recombinant proteins and antibodies

Several recombinant His-tagged proteins, including prefusion-stabilized SARS-CoV-2 S-trimer protein, SARS-CoV-2 RBD protein, SARS-CoV RBD protein, and hACE2-hFc fusion protein (human ACE2 extracellular domain fused with human IgG1 Fc) were generated in a previous study [37]. To prepare prefusion-stabilized S proteins of SARS-CoV-2 variants including B.1.1.7, B.1.351, B.1.617.1, B.1.617.2, codon-optimized genes encoding S ectodomain with a “GSAS” substitution at the furin cleavage site and a double proline substitution [10,39] was cloned into vector pcDNA 3.1+ with a C-terminal T4 fibritin trimerization motif, a TEV protease cleavage site, a FLAG tag and a 9×His tag, yielding the corresponding expression vectors (eg. pcDNA 3.1-B.1.351-S). To prepare SARS-CoV-2 NTD, the DNA fragment corresponding to the NTD region (residues V16 to D294) of SARS-CoV-2 strain Wuhan-Hu-1 (GenBank ID: MN908947.3) was codon optimized and inserted into a modified pcDNA3.4 vector that contains an N-terminal interleukin-10 (IL-10) signal sequence and a C-terminal 6×His tag, yielding plasmid pcDNA3.4-NTD. These constructs were separately transfected into HEK 293F suspension cells and the resulting culture supernatants containing His-tagged proteins were subjected to purification using Ni-NTA resin (Millipore, USA) according to manufacturer's protocol.

A polyclonal antibody against recombinant SARS-CoV-2 RBD was produced in our previous study [37]. Anti-SARS-CoV-2 mouse MAbs 2H2 and 3C1 were generated in our previous study [37]. MAb 5F8 is a murine IgG antibody against E protein of zika virus (ZIKV) [40] and was used as isotype control.

### Isolation of anti-SARS-CoV-2 MAbs from immunized mice

The animal studies were approved by the Institutional Animal Care and Use Committee at the Institut Pasteur of Shanghai.

To prepare anti-S MAbs, adult female BALB/c mice were each immunized intraperitoneally (i.p.) with 50 μg wild-type S-trimer protein in combination with 0.5 mg aluminum hydroxide adjuvant (alum; Invivogen, USA) and 25 μg CpG oligonucleotides on day 0. Each mouse was boosted i.p. on day 14 with 50 μg S-trimer emulsified with Freund's complete adjuvant (Sigma, USA), on day 28 with 50 μg S-trimer in combination with 0.5 mg alum and 25 μg CpG, and on day 35 with 50 μg S-trimer emulsified with Titermax adjuvant (Sigma). On day 42 one mouse was selected and injected with 100 μg S-trimer in PBS via the tail vein. On day 46 the mouse splenocytes were used to generate hybridomas using our previously reported protocol [41]. 12 days later, hybridoma supernatants were screened by pseudovirus neutralization assay as described below. Neutralization-positive hybridoma cells were cloned by limiting dilution, resulting in monoclonal cell lines. Isotypes of the MAbs were determined using the SBA Clonotyping system/HRP kit (Southern Biotech, USA) according to manufacturer's instructions. Immunoglobulin variable-region genes were amplified and sequenced using degenerate primers as described previously [37]. Antibody sequence analysis was performed using IgBLAST [42]. MAbs were purified using protein G agarose resin 4FF (Yeasen, China) according to our previously described protocol [43].

### Murine leukemia virus (MLV)-based pseudovirus neutralization assay

MLV-based SARS-CoV-2 S pseudoviruses were prepared according to a previously reported protocol [37], except that plasmids encoding full-length S protein of SARS-CoV-2 prototype strain Wuhan-Hu-1, B.1.1.7, B.1.351, B.1.617.1 or B.1.617.2 variants were used. Similarly, MLV-based SARS-CoV S pseudoviruses were prepared using the plasmid pHCMV-SARS-S (FFM-1) (GenBank ID: AAS75868) [44].

Pseudovirus neutralization assay was performed in 96-well plates with 293T-hACE2 cells as described previously [37]. At 48 h post infection, luciferase activity was measured by luciferase assay system (Promega). Percent neutralization was calculated by the following equation: [1 − (luminescence of sample − luminescence of the cell-only control) / (luminescence of the pseudovirus-only control – luminescence of the cell-only control)] × 100%. For each MAb, half inhibitory concentration (IC50) was calculated using GraphPad Prism software by nonlinear regression.

### Authentic virus neutralization assay

The live SARS-CoV-2 infection/neutralization experiments were carried out in the biosafety level-3 (BSL-3) laboratory of Fudan University according to a protocol described previously [37]. Two days after infection, culture supernatants were harvested for reverse transcription quantitative PCR (RT-qPCR) determination of viral RNA levels, and cells were fixed for immunofluorescence analysis.

### Binding ELISA

To assess the binding properties of the MAbs, ELISA plates were coated with 100 ng/well of S-trimer, RBD, or NTD at 4°C overnight and then blocked with 5% milk in PBS-Tween20 (PBST). After washing, the plates were incubated with 100 ng/well of the MAbs for 2 h at 37°C, followed by incubation with horseradish peroxidase (HRP)-conjugated anti-mouse IgG (Sigma, diluted 1:10,000) for 1 h at 37°C. After washing and colour development, absorbance was monitored at 450 nm.

### Binding affinity determination by bio-layer interferometry (BLI)

To determine the RBD-binding affinities of the MAbs, BLI experiments were carried out on an Octet Red96 instrument (Pall FortéBio, USA). Wild-type RBD or S trimer protein was loaded onto the Ni-NTA biosensors (Pall FortéBio) until saturation. The antigen-coated sensors were moved to wells containing individual MAb samples at varying concentrations for a 500-sec association step and then moved to wells containing dissociation buffer (0.01 M PBS with 0.02% Tween 20 and 0.1% bovine serum albumin) for a 500-sec dissociation step. Data were analyzed using Octet data analysis software version 11.0 (Pall FortéBio).

### Mapping of MAb epitopes with RBD mutants by ELISA

For epitope mapping, RBD mutants, including mutants RBD (Core), RBD (RBM-R2), and RBD (RBM-R3) generated in a previous study [37], were constructed using the MutExpress II Fast Mutagenesis Kit V2 (Vazyme, China) according to the manufacturer’s instruction. These mutant RBD proteins were produced using HEK293F expression system and purified using Ni-NTA resin as described above.

The RBD mutants were evaluated for binding to the MAbs by ELISA. Briefly, ELISA plates were coated with wild-type RBD protein or individual RBD mutants (200 ng/well) in PBS at 4°C overnight and then blocked. Next, the plates were incubated with anti-RBD sera (diluted 1:1000) or individual MAbs (50 ng/well) at 37°C for 2 h, followed by incubation with HRP-conjugated anti-mouse IgG (Sigma; diluted 1:10,000). After colour development, absorbance at 450 nm was measured.

### Receptor competition ELISA assay

Microplates were coated at with 40 ng/well of wild-type S-trimer or RBD at 4°C overnight followed by blocking. After washing, diluted purified MAbs (25 μL) were mixed with 20 ng (25 μL) of biotinylated ACE2-hFc prior to addition to the wells. After incubation at 37°C for 2 h and washing, HRP-conjugated streptavidin (Life Technologies, USA; diluted 1:2000) was added and incubated at 37°C for 1 h. After washing and colour development, absorbance was monitored at 450 nm.

### BLI-based antibody competition assay

Ni-NTA biosensors were coated with WT RBD. Next, the antigen-coated sensors were allowed to react with 100 nM of the first antibody followed by incubation with 100 nM of a second MAb. Data were analyzed using Octet data analysis software version 11.0.

### Vesicular stomatitis virus (VSV)-based pseudovirus neutralization assay

A replication-competent VSV bearing SARS-CoV-2 S protein (VSV-S) and GFP reporter was generated using the previously reported protocol [45] and then propagated in VeroE6-hACE2 cells.

Before neutralization assay, each well of the 96-well plates was seeded with 16,000 VeroE6-hACE2 cells and incubated overnight. Next, 100 TCID_50_ of VSV-S was mixed with serially diluted MAbs, and the mixtures were then added to the wells. After culture at 37°C for 36 h, the culture supernatant was removed, and the cells were fixed in 100 μL of 4% paraformaldehyde. Subsequently, fluorescent foci were counted using Cytation 5 microplate reader (BioTek, USA). Percent neutralization was calculated relative to the VSV-S only wells.

### Screening of neutralization-resistant mutants

To obtain escape variants, the replication-competent VSV-S was passaged under increasing concentrations of MAb 5D2 or 5G2. The resulting escape mutants were purified by one round of plaque purification followed by propagation and sequencing. Individual escape mutants were titrated and subsequently evaluated for resistance to neutralization by a given MAb (1.5 and 12.5 μg/mL) by neutralization assay as described above. The images of green fluorescence were taken using Cytation 5 microplate reader.

### SARS-CoV-2 B.1.351 variant S trimer/Fab complex formation

To generate related Fabs, purified MAbs was incubated with papain (300:1 W/W) in PBS buffer (in the presence of 20 mM L-cysteine and 1 mM EDTA) for 3 h at 37°C. The reaction was quenched by 20 mM iodoacetamide. Fab was purified by running over a HiTrap DEAE FF column (GE Healthcare) pre-equilibrated with 10 mM PBS. SARS-CoV-2 B.1.351 S protein was incubated with individual Fab in a molar ratio of 1:4 or 1:8 on ice for 1 h. The S-Fab complex was purified by size-exclusion chromatography using Superose 6 increase 10/300 GL column (GE Healthcare) in 20 mM Tris-HCl pH 7.5, 200 mM NaCl, 4% glycerol. The complex peak fractions were concentrated and assessed by SDS-PAGE (Supplementary Figure 7).

### Cryo-EM sample preparation and cryo-EM data collection

An aliquot (2.2 μL) of the B.1.351 S-S5D2 Fab sample was applied on a plasma-cleaned holey carbon grid (R1.2/1.3, 200 mesh; Quantifoil). The grid was blotted with Vitrobot Mark IV (Thermo Fisher Scientific) and then plunged into liquid ethane cooled by liquid nitrogen. The above-mentioned procedure was followed to prepare the vitreous sample for the B.1.351 S-S3H3 complex.

Cryo-EM movies for the samples were collected on a Titan Krios electron microscope (Thermo Fisher Scientific) operated at an accelerating voltage of 300 kV with a magnification of 64,000×. The movies were recorded on a Gatan K3 direct electron detector operated in the counting mode (yielding a pixel size of 1.093 Å) under a low-dose condition in an automatic manner using EPU software (Thermo Fisher Scientific). Each frame was exposed for 0.1 s, and the total accumulation time was 3 s, leading to a total accumulated dose of 50 e^−^/Å^2^ on the specimen (Supplementary Table S2).

### Cryo-EM data processing and structure refinement

The motion correction of each image stack was performed using the embedded module of Motioncor2 [46] in Relion 3.1 [47] and CTFFIND4 was used to determine CTF parameters before further data processing [48]. Single-particle analysis was mainly executed in Relion 3.1 and cryoSPARC [49]. After automatic particle picking, manual selection, and multiple rounds of reference-free 2D classification, cleaned up particles remained for further reconstruction with our previous SARS-CoV-2 S-open cryo-EM map (EMDB:30701) as an initial model [11]. For the S-S5D2 Fab complex, after multiple rounds of 2D and 3D classifications, we obtained a S-S5D2 Fab map from 199,785 cleaned-up particles. After CTF refinement and Bayesian polishing, the S-S5D2 map was refined to 3.2-Å-resolution. Here, to improve the interface between RBD and S5D2 Fab, we subtracted the more steady RBD-1-Fab region and performed 3D classification and local refinement in cryoSPARC, resulting in a 3.5-Å-resolution RBD1-Fab map from 100,064 particles. Moreover, for the 3.2-Å-resoution S-S5D2 map, we performed further focused 3D classification on the relative weak RBD-2-S5D2 Fab and RBD-3-S5D2 Fab regions. Eventually, we obtained a S-S5D2-F1 map from 35,322 particles, a S-S5D2-F2 map from 129,238 particles, and a S-S5D2-F3 map from 35,225 particles at 3.5, 3.3, and 3.5 Å resolution, respectively. The overall resolution was determined on the basis of the gold standard criterion using a Fourier shell correlation (FSC) of 0.143. For the B.1.351 S-S3H3 complex, similar data processing procedure was adapted as described above.

### Pseudo atomic model building

The homology models of the S5D2 and S3H3 Fab were built through SWISS-MODEL webserver [50]. For the S protein portion in the S-S5D2 Fab and S-S3H3 Fab structures, we used the available open state model of the SARS-CoV-2 S trimer (PDB: 7DF4) [11] as initial model and substituted the B.1.351 variations using COOT software package [51]. We then fitted the models of individual protomer/Fab into the corresponding region in the related density map as rigid body using UCSF Chimera [52], and combined them into a complete model. Subsequently, we refined each of the models against corresponding cryo-EM density map using Rosetta [53] then Phenix [54]. The final pseudo atomic models were validated using Phenix.molprobity command in Phenix. UCSF Chimera and ChimeraX were used for map segmentation and figure generation [52,55].

### Statistical analysis

All statistical analyses were performed using GraphPad Prism version 7. Nonlinear regression was analyzed by Variable Slope (Four Parameters) and the fitting method used was least squares (ordinary) fit**.**

## Results

### Isolation and classification of SARS-CoV-2 neutralizing MAbs

**Figure 1.**
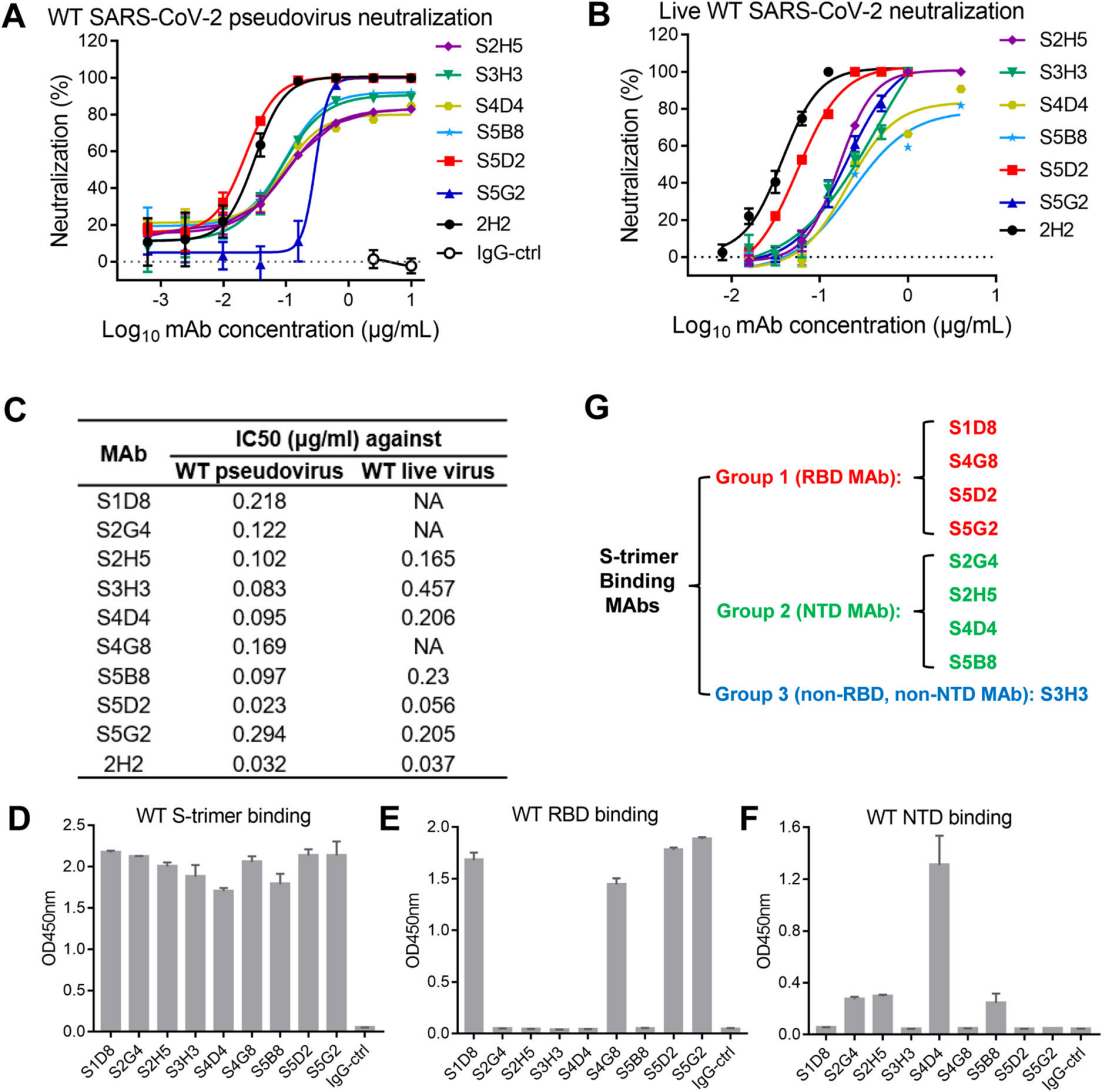
Neutralization activity and binding properties of anti-SARS-CoV-2 MAbs. (A) Representative neutralization curves of the MAbs against wild-type (WT) SARS-CoV-2 pseudovirus. Zika virus (ZIKV) MAb 5F8 was used as IgG isotype control (IgG-ctrl) in all experiments. Data are expressed as mean ± SD of four replicate wells. (B) Live SARS-CoV-2 virus neutralization determined by real-time RT-PCR. Data are mean ± SD of three replicate wells. (C) Summary of the IC50s of the 9 neutralizing MAbs against SARS-CoV-2 WT pseudovirus or authentic virus. For panels A-C, NA, not analyzed. 2H2 is a previously identified neutralizing antibody, serving as a reference antibody in this study. (D-F) Binding activities of the MAbs to recombinant SARS-CoV-2 WT S-trimer (D), RBD protein (E), and NTD protein (F) were determined by ELISA. Data are expressed as mean ± SD of triplicate wells. (G) Grouping of the MAbs. Antibody binding targets were shown in brackets.

We generated a series of SARS-CoV-2 neutralizing MAbs from mice immunized with the recombinant trimeric S protein (S-trimer) of an original wild-type (WT) SARS-CoV-2 strain (Wuhan-Hu-1) by using conventional hybridoma technology. Screening of hybridoma supernatants for neutralization of WT SARS-CoV-2 pseudovirus revealed that a total of 9 clones were neutralizing, designated S1D8, S2G4, S2H5, S3H3, S4D4, S4G8, S5B8, S5D2, and S5G2, respectively (Supplementary Table S1). Among the 9 clones, S5G2 was found to be able to cross-neutralize SARS-CoV pseudovirus (Supplementary Table S1). Variable region sequence analysis verified that these 9 hybridomas are distinct cell clones (Supplementary Figure 1A). Isotyping analysis showed that clone S5G2 is IgG2b and the others belong to IgG1 (Supplementary Table S1). Purified MAbs were assessed for their neutralization potency against WT SARS-CoV-2 pseudovirus. All of the MAbs exhibited potent neutralization activity with half inhibitory concentrations (IC50) ranging from 0.023 to 0.294 µg/mL (Figure 1(A, C)). Among the 9 MAbs, S5D2 appeared to be the strongest neutralizer, whose neutralization potency was comparable to that of the antibody 2H2 (Figure 1(A, C)), a highly neutralizing MAb identified in our previous study [37].

MAbs S2H5, S3H3, S4D4, S5B8, S5D2, and S5G2 were further assessed for neutralization of authentic SARS-CoV-2 (nCoV-SH01 strain) infection of VeroE6 cells. Results from both immunostaining and qRT-PCR assays showed that these mAbs dose-dependently neutralized authentic SARS-CoV-2 with IC50s ranging from 0.056 to 0.457 µg/mL and S5D2 exhibited the highest potency (IC50 = 0.056 µg/mL) (Figure 1(B, C) and Supplementary Figure 2). These results were in line with the data from pseudovirus neutralization assays, thus confirming the neutralization potency of the MAbs.

We then assessed the binding properties of the MAbs by ELISA and bio-layer interferometry (BLI) assay. As expected, all of the 9 MAbs were capable of binding to recombinant SARS-CoV-2 S trimer protein in ELISA (Figure 1(D)). However, when recombinant RBD protein was used as the capture antigen, only MAbs S1D8, S4G8, S5D2, and S5G2 showed strong binding while the other MAbs did not exhibit any reactivity (Figure 1(E)). All of MAbs S1D8, S4G8, S5D2, and S5G2 had binding affinity (KD) values of less than 1 pM towards SARS-CoV-2 RBD (Supplementary Figure 3(A, B)). The 9 MAbs were then tested for binding to recombinant NTD protein by ELISA. The results showed that only MAbs S2G4, S2H5, S4D4, and S5B8 could bind to NTD (Figure 1(F)). Interestingly, MAb S3H3 did not show any reactivity with monomeric RBD or NTD in ELISA despite it could efficiently bind S-trimer (Figure 1(D, E, F)), suggesting that S3H3 may target an epitope located outside of the NTD and RBD regions on S protein or only recognize a quaternary epitope that requires trimeric configuration. Based on the above binding data, we divided the 9 neutralizing MAbs into three groups: group 1 consists of S1D8, S4G8, S5D2, and S5G2, all of which target the RBD region; group 2 is comprised of S2G4, S2H5, S4D4, and S5B8, all of which bind NTD; group 3 contains only S3H3, which is a non-RBD- and non-NTD-reactive antibody (Figure 1(G)).

### Cross-neutralization capacity of the MAbs towards major SARS-CoV-2 variants

**Figure 2.**
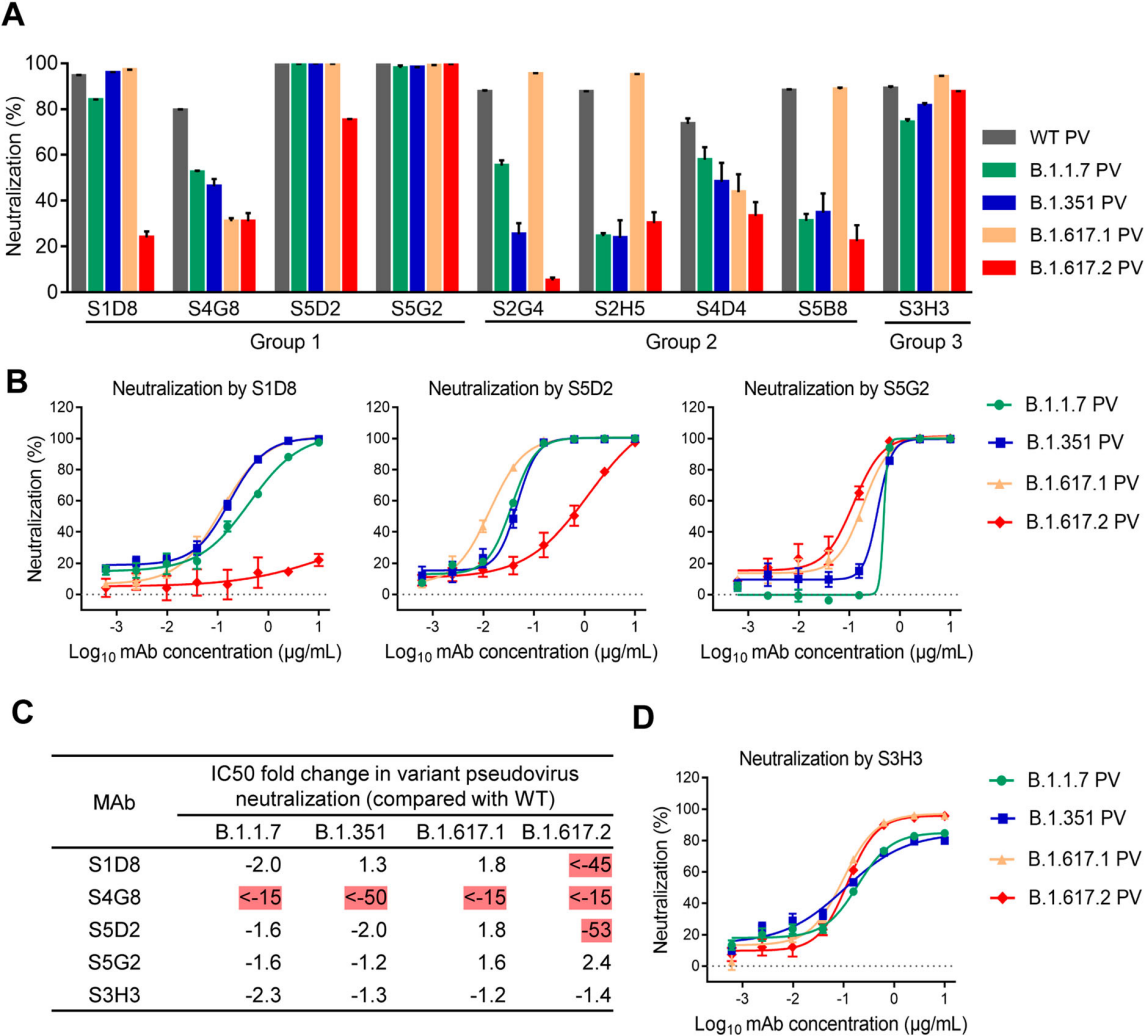
Neutralization breadth of the MAbs against SARS-CoV-2 variants. (A) Neutralization activity of the MAbs against SARS-CoV-2 wild-type (WT), B.1.1.7, B.1.351, B.1.617.1 and B.1.617.2 variant pseudoviruses. For each MAb, a fixed concentration (1 μg/mL) was tested for neutralization of murine leukemia virus (MLV) pseudotyped with SARS-CoV-2 spike protein. Data are expressed as mean ± SEM of triplicate wells. Results shown are representative of two independent experiments. (B) Neutralization curves of MAbs S1D8, S5D2 and S5G2 against the variant pseudoviruses. MAbs were four-fold serially diluted and subjected to pseudovirus neutralization assay. (C) Fold increase or decrease in IC50s of neutralizing MAbs against B.1.1.7, B.1.351, B.1.617.1, and B.1.617.2 pseudoviruses relative to the WT pseudovirus, Red, resistance >2.5-fold. (D) Neutralization curves of S3H3 against the variant pseudoviruses. For panels B and D, data are expressed as mean ± SEM of four replicate wells and results shown are representative of two independent experiments.

For assessment of neutralization breadth of our MAbs, we generated a panel of pseudoviruses representing major circulating SARS-CoV-2 variants, including B.1.1.7, B.1.351, B.1.617.1, and B.1.617.2 (Supplementary Figure 4). The 9 MAbs were tested in parallel for neutralization of the variant pseudovirus panel as well as the WT pseudovirus (Figure 2(A)). We firstly used a fixed antibody concentration (1 μg/mL) for all MAbs to screen the pseudovirus panel, and after identifying broad-spectrum neutralizer, we further determined their IC50s against each of the pseudoviruses.

Among the group 1 MAbs, S1D8 and S5D2 exhibited comparable neutralization potency towards B.1.1.7, B.1.351, and B.1.617.1 variants, relative to the WT strain, but showed drastically reduced neutralizing activity against B.1.617.2 (Figure 2(A, B, C, D)). Specifically, the IC50s of S5D2 towards B.1.1.7, B.1.351, B.1.617.1, and B.1.617.2 were determined to be 0.037, 0.047, 0.013, and 1.219 μg/mL, respectively; and the corresponding ones for S1D8 were 0.430, 0.174, 0.120, and >10 μg/mL, respectively. S4G8 displayed significantly reduced neutralizing activity against each of the four variants relative to the WT strain (Figure 2(A, C)). In contrast, S5G2 could broadly neutralize the four variants with comparable IC50s (less than 2.5-fold change relative to the WT pseudovirus) (Figure 2(A, C)). These data reveal diverse neutralization spectrums for the RBD-targeting MAbs.

For all of the group 2 antibodies, their neutralization potency towards B.1.1.7, B.1.351, and B.1.617.2 showed an overall trend of decreasing, relative to that against the WT pseudovirus (Figure 2(A)). Compared to the WT pseudovirus, B.1.617.1 variant exhibited similar sensitivity to MAbs S2G4, S2H5, and S5B8, but showed increased resistance to S4D4. These data indicated that none of the NTD-targeting MAbs was broadly neutralizing.

S3H3, the sole member in the group 3, was found to retain neutralizing potency towards all the four variants tested (Figure 2(C)). Specifically, S3H3 effectively neutralized B.1.1.7, B.1.351, B.1.617.1, and B.1.617.2 pseudoviruses with IC50s of 0.191, 0.107, 0.098, and 0.119 μg/mL, respectively (Figure 2(D)), comparable to that against the WT pseudovirus (0.083 μg/mL) (Figure 1(B)). Consistently, S3H3 showed similar binding affinities to the S-trimers derived from the WT or variant strains (Supplementary Figure 3C). These data indicate that S3H3 possesses cross-variant binding and neutralizing abilities.

### Epitope mapping of the group 1 MAbs

**Figure 3.**
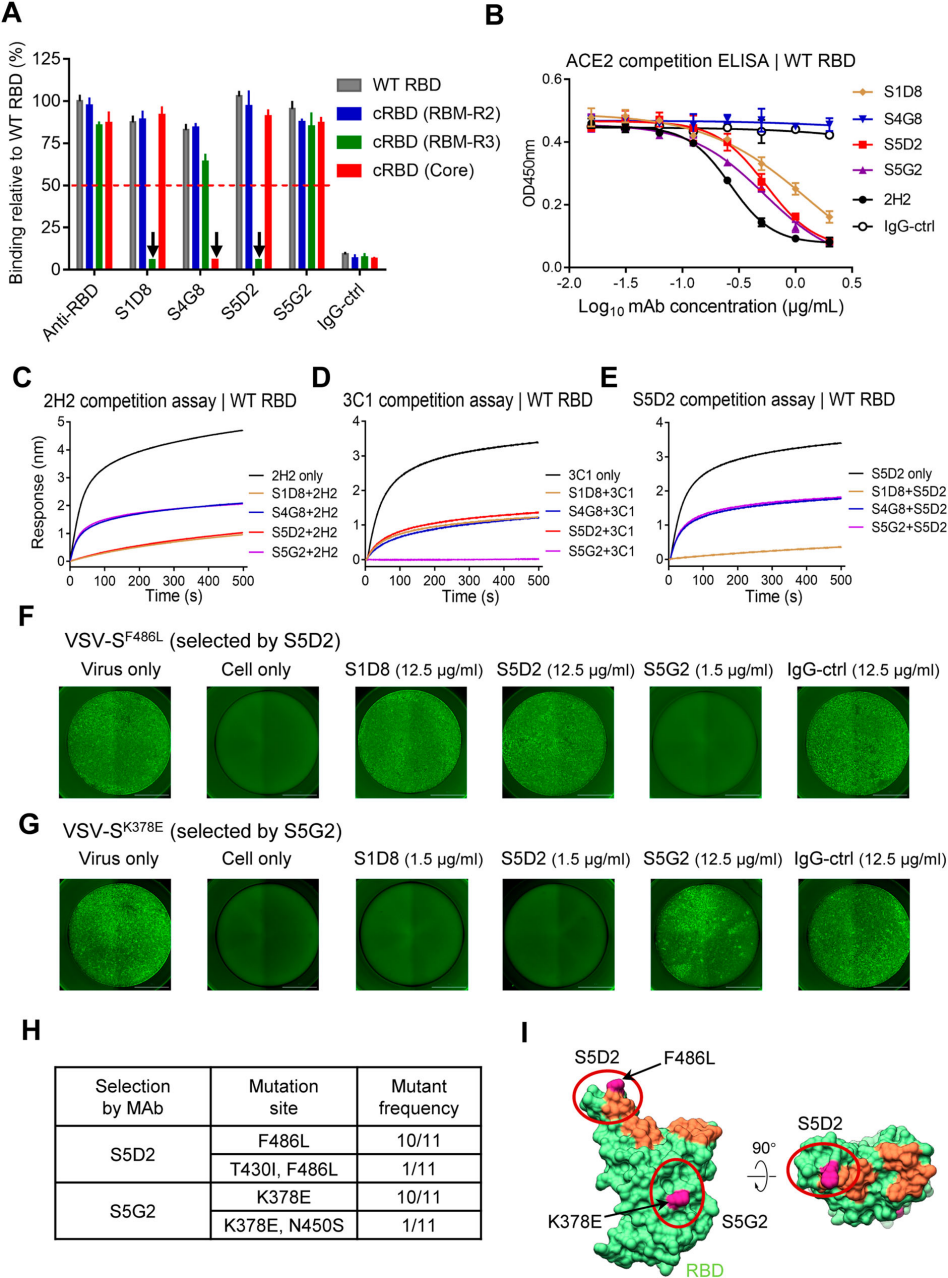
Epitope mapping for the group 1 MAbs. (A) Binding activities of the MAbs to WT and chimeric RBD proteins(cRBD) were determined by ELISA. For cRBD(RBM-R2), cRBD(RBM-R3), and cRBD(Core), residues L452-K467, T470-T478, and R319-N437 in the SARS-CoV-2 RBD were separately replaced by the counterpart from SARS-CoV. Anti-RBD polyclonal antibody served as positive control. The downward arrow indicates that substitutions in RBD mutants significantly reduced the binding of the MAbs compared to WT RBD. Binding level of anti-RBD polyclonal antibody to WT RBD was set to 100%, and red dotted line represents cutoff value (50%). IgG-ctrl, anti-ZIKV MAb 5F8. Data are mean ± SD of triplicate wells. (B) Competition between the MAbs and ACE2 for binding to WT RBD was determined by ELISA. The ACE2-binding signal was detected by a corresponding secondary antibody. Data are mean ± SD of triplicate wells. (C–E) MAb competition determined by BLI. Immobilized RBD was first saturated with an MAb as indicated and then allowed to interact with MAb 2H2 (C), 3C1 (D), or S5D2 (E). The resulting binding signals of 2H2, 3C1 and S5D2 were shown. Binding of RBD with 2H2, 3C1, or S5D2 alone was used as reference control. (F–G) Escaping mutants selected with the antibody S5D2 (F) or S5G2 (G) by using reporter VSV pseudotyped with SARS-CoV-2 WT spike and tested for resistance to the MAbs. Antibody concentration used was shown in parentheses. (H) Mutation site and frequency of mutants selected by antibody S5D2 and S5G2. (I) Predicted interaction regions of S5D2 and S5G2 on RBD. The area in coral shows the ACE2 binding site on RBD.

To roughly locate the epitopes of the group 1 MAbs, we tested these MAbs for binding with a panel of three chimeric RBD mutants developed in our recent study [37]. These RBD mutants, designated cRBD (Core), cRBD (RBM-R2) and cRBD (RBM-R3), were generated by displacing the RBD core region (R319 to N437), and the RBM R2 (L452 to K462) and R3 (T470 to T478) regions of SARS-CoV-2 with the counterparts of SARS-CoV, respectively [37]. As shown in Figure 3A, MAb S1D8 effectively bound with the cRBD (RBM-R2) and cRBD (Core) mutants as well as the WT RBD, but failed to recognize the cRBD (RBM-R3) mutant; similar binding pattern was also observed for MAb S5D2. These results indicate that S1D8 and S5D2 target the R3 region of RBM. MAb S4G8 strongly reacted with the cRBD (RBM-R2) and cRBD (RBM-R3) mutants but not with cRBD (Core), suggesting that S4G8 may target the core region of RBD. MAb S5G2 reacted strongly with all of the chimeric RBD mutants, indicating that S5G2 recognizes a conserved epitope shared by SARS-CoV and SARS-CoV-2.

We then determined whether these MAbs could compete with ACE2 receptor for WT SARS-CoV-2 RBD binding by performing competition ELISA. The previously identified MAb 2H2, which binds the RBM region [37], efficiently blocked ACE2 binding to the WT RBD, thus validating the assay (Figure 3(B) and Supplementary Figure 3D). MAbs S1D8, S5D2, and S5G2 were able to inhibit ACE2 binding to the RBD in a dose-dependent manner, whereas MAb S4G8 did not show any inhibitory effect (Figure 3(B) and Supplementary Figure 3D). The results suggest that the epitopes of S1D8, S5D2, and S5G2 overlap with the ACE2-binding site (also known as RBM) whereas the S4G8 antibody epitope is likely away from the RBM.

We then performed BLI assay to evaluate binding competition of each of these MAbs against two well-characterized MAbs, 2H2 and 3C1, which bind to the top (RBM) and the side (core) of RBD, respectively [37]. In this assay, immobilized WT RBD protein was preincubated with each of the group 1 MAbs prior to interacting with 2H2 or 3C1 antibodies. Compared with 2H2 alone, preincubation with MAbs S1D8, S4G8, S5D2 or S5G2 reduced subsequent 2H2 binding to varying degrees (Figure 3(C)). Particularly, pretreatment with S1D8 or S5D2 resulted in drastic decrease in 2H2 binding signal, suggesting that the epitopes of these two MAbs may largely overlap with that of MAb 2H2. In the 3C1 binding competition assay, MAbs S1D8, S4G8, and S5D2 exhibited partial inhibition on 3C1 binding (Figure 3(D)). Of note, MAb S5G2 completely blocked the binding of 3C1 to RBD (Figure 3(D)), suggesting that the epitope of S5G2 is similar to or highly overlaps with the 3C1 epitope that involves mainly the β2-strand (T376 to C379) and loop^380–385^ in the core region of RBD and is conserved between SARS-CoV-2 and SARS-CoV [37] (Supplementary Figure 5). Consistently, we found that MAb S5G2 was able to efficiently bind recombinant SARS-CoV RBD protein with KD < 1 pM and potently cross-neutralize SARS-CoV pseudovirus with IC50 of 0.071 μg/mL (Supplementary Figure 5).

The above biochemical analyses suggest that the group 1 MAbs could be further divided into three subgroups: subgroup 1a comprises S5D2 and S1D8 which target primarily the RBM and block ACE2 binding; subgroup 1b consists of only S5G2, binding mainly the RBD core and exhibiting ACE2 blockade probably through steric hindrance; and subgroup 1c contains only S4G8 which also recognizes the core region of RBD but has no ACE2-blocking activity. BLI assays showed that S1D8 almost completely abolished S5D2 binding with the WT RBD (Figure 3(E)), suggesting that S1D8 and S5D2 may recognize the same antigenic site.

We also selected antibody-escape mutants to identify critical residues within the binding epitope of MAbs S5D2 and S5G2 by using a vesicular stomatitis virus (VSV) pseudovirus system bearing SARS-CoV-2 S protein (Supplementary Figure 6A). The resulting escape mutants were plaque-purified and sequenced (Figure 3(F, G, H)). Among 11 recovered S5D2-resistant mutants, 10 had a single amino acid change (Phe to Leu) at residue 486 (F486L) while the remaining one contained not only the F486L change but also an additional mutation at residue 430 (T430I). The S5D2-escaping virus harbouring the single F486L mutation (designated VSV-S^F486L^) was resistant to its selecting antibody S5D2 (neutralization concentration >12.5 μg/mL) but remained sensitive to MAb S5G2 (Figure 3(F)), thus revealing F486, residing within the RBM, as a critical residue of the S5D2 epitope. In addition, the VSV-S^F486L^ virus also showed resistance to high concentration (12.5 μg/mL) of S1D8 (Figure 3(F)), confirming that S5D2 and S1D8 may share the same or highly similar epitope. Under S5G2 selection, 11 resistant virus clones were obtained, among which 10 clones possessed a Lys to Glu change at residue 378 (K378E) and the remaining one had an additional mutation at residue 450 (N450S) besides K378E (Figure 3(H)). The S5G2-escaping virus carrying a single K378E mutation (designated VSV-S^K378E^), was refractory to neutralization by its selecting antibody S5G2 (neutralization concentration >12.5 μg/mL) but remained sensitive to S5D2 (Figure 3(G)). These results demonstrate that K378, located within the core region of RBD (Figure 3(I)), is essential for S5G2 binding, and therefore confirm that S5G2 binds the RBD core region.

Taken together, the above data reveal that: (1) The epitopes of both S1D8 and S5D2 MAbs highly overlap with the ACE2 binding site and involve mainly the RBM R3 region (T470 to T478) and also the residue F486 (Figure 3(I)); (2) the S4G8 epitope resides within the RBD core region and has no overlap with ACE2 binding site; (3) MAb S5G2 recognizes a conserved epitope highly similar to that of MAb 3C1 which mainly consists of residues T376 to T385 of RBD core region [37].

### Cryo-EM structures of the B.1.351 S trimer in complex with S5D2 Fab

**Figure 4.**
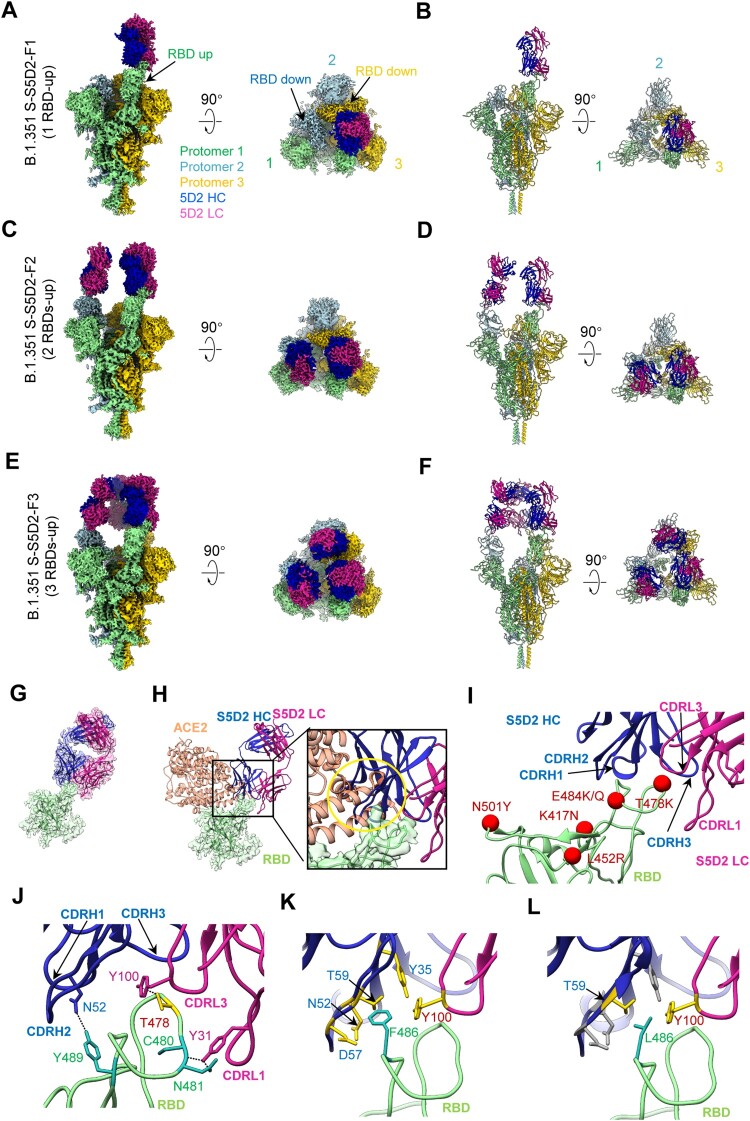
Cryo-EM structures of the SARS-CoV-2 B.1.351 S trimer in complex with S5D2 Fab. (A and B) Side and top views of the B.1.351 S-S5D2-F1 cryo-EM map (A) and pseudo atomic model (B). Only the RBD-1 is in up configuration, which binds with a S5D2 Fab. Protomer 1, 2, and 3 are shown in light green, light blue, and gold, respectively. This colour scheme is followed throughout. Heavy chain and light chain of S5D2 Fab is in medium blue and violet red, respectively. (C and D) Side and top views of the S-S5D2-F2 cryo-EM map (C) and pseudo atomic model (D), with two up RBDs (RBD-1 and RBD-2) each bound with a S5D2 Fab. (E and F) Side and top views of the S-S5D2-F3 cryo-EM map (E) and pseudo atomic model (F), with three up RBDs each bound with a S5D2 Fab. (G) Local refined RBD-1-S5D2 structure. (H) S5D2 Fab and ACE2 (coral, PDB: 6M0J) share overlapping epitopes on RBD and would clash upon binding to the S trimer. Gold circle indicates clashed area. (I) S5D2 Fab binds to the RBD (light green), with major involved structural elements labelled. The mutated amino acids of main variants are marked in red. (J) The involved regions/residues forming hydrogen bond between S5D2 Fab and RBD-1. (K and L) The contact network was altered due to the RBD F486L mutation (in light sea green).

Among the group 1 neutralizing MAbs, S5D2 exhibited highest neutralization potency against the WT strain and the variants (except B.1.617.2). To investigate the molecular basis of S5D2-mediated cross-variant neutralization, we performed cryo-EM single particle analysis to determine the structures of SARS-CoV-2 B.1.351 S trimer in complex with S5D2 Fab (Supplementary Figures 8 and 9). Three cryo-EM maps, including the SARS-CoV-2 B.1.351 S-S5D2-F1 (associated with one Fab), S-S5D2-F2 (with two Fabs), and S-S5D2-F3 (with three Fabs) states were resolved to 3.5, 3.3, and 3.5 Å resolution, respectively (Figure 4(A-F); Table S2; Supplementary Figure 8 and 9). These maps show that only the “up” RBDs can bind with S5D2 Fab, while the remaining RBDs are in the “down” conformation without Fab binding (Figure 4(A–F)).

To better investigate the interaction between S5D2 Fab and the S protein of the B.1.351 variant, we performed additional focused 3D-classification and refinement on the RBD-1-Fab region, and obtained an RBD-1-S5D2 map with improved local resolution (Figure 4(G); Supplementary Figures 8 and 9(C, D)). Our structural study showed that S5D2 Fab binds on the top of the up RBD (Figure 4(A–F)), which also mediates the binding of the WT S with human ACE2 receptor (Figure 4(H)). The epitope of S5D2 Fab on RBD would partially overlap with the binding sites of ACE2 on RBD, leading to clash and spatial hindrance between ACE2 and the S5D2 Fab (Figure 4(H)). This is in line with the high potency of S5D2 on blocking the interaction between RBD and ACE2 (Figure 3(B)). The better resolved RBD-1-S5D2 structure reveals that the CDRL1 and CDRL3, together with all the three heavy-chain CDRs of S5D2 form contacts with the RBD (Figure 4(I, J)). Specifically, the Y31 of CDRL1 and Y100 of CDRL3 form hydrogen bond with the C480, N481, and T478 of RBD, respectively; in addition, the N52 of CDRH2 also forms hydrogen bond with the Y489 of RBD (Figure 4(J)). These hydrogen bonds and the close contacts (with 4-Å cutoff) constitute an interaction network, with the pocket formed by the S5D2 Fab tightly wrapping around the RBM loop^477–489^. Besides, F486, also belonging to the RBM loop^477–489^, form contacts with up to five residues (Y35, N52, D57, T59 of CDRH, and Y100 of CDRL) of S5D2 Fab, formulating a key interaction site (Figure 4(K) and Supplementary Table 3). When F486 was substituted to L with smaller side chain, it led to the breakage of the contact network with only two contacting residues (T59 and Y100) remained (Figure 4(L)). This explains why the F486L mutant virus could escape S5D2 neutralization (Figure 3(F)).

Our structures suggested that the mutations on the B.1.351 RBD, including K417N, E484 K, and N501Y, are not the contact residues of S5D2 Fab and will not affect binding of S5D2 on the S trimer (Figure 4(I, J)). This explains why S5D2 exhibited strong binding affinity to the S trimer of both the original strain and the B.1.351 variant. As we also showed in Figure 4I, for the B.1.1.7 (N501Y mutation on RBD) and B.1.617.1 (L452R and E484Q mutations on RBD) variants, their mutations are not involved in the RBD-S5D2 interaction interface and will not affect the efficacy of the S5D2 towards these variants.

### A unique epitope for broadly neutralizing antibody S3H3 revealed by cryo-EM

**Figure 5.**
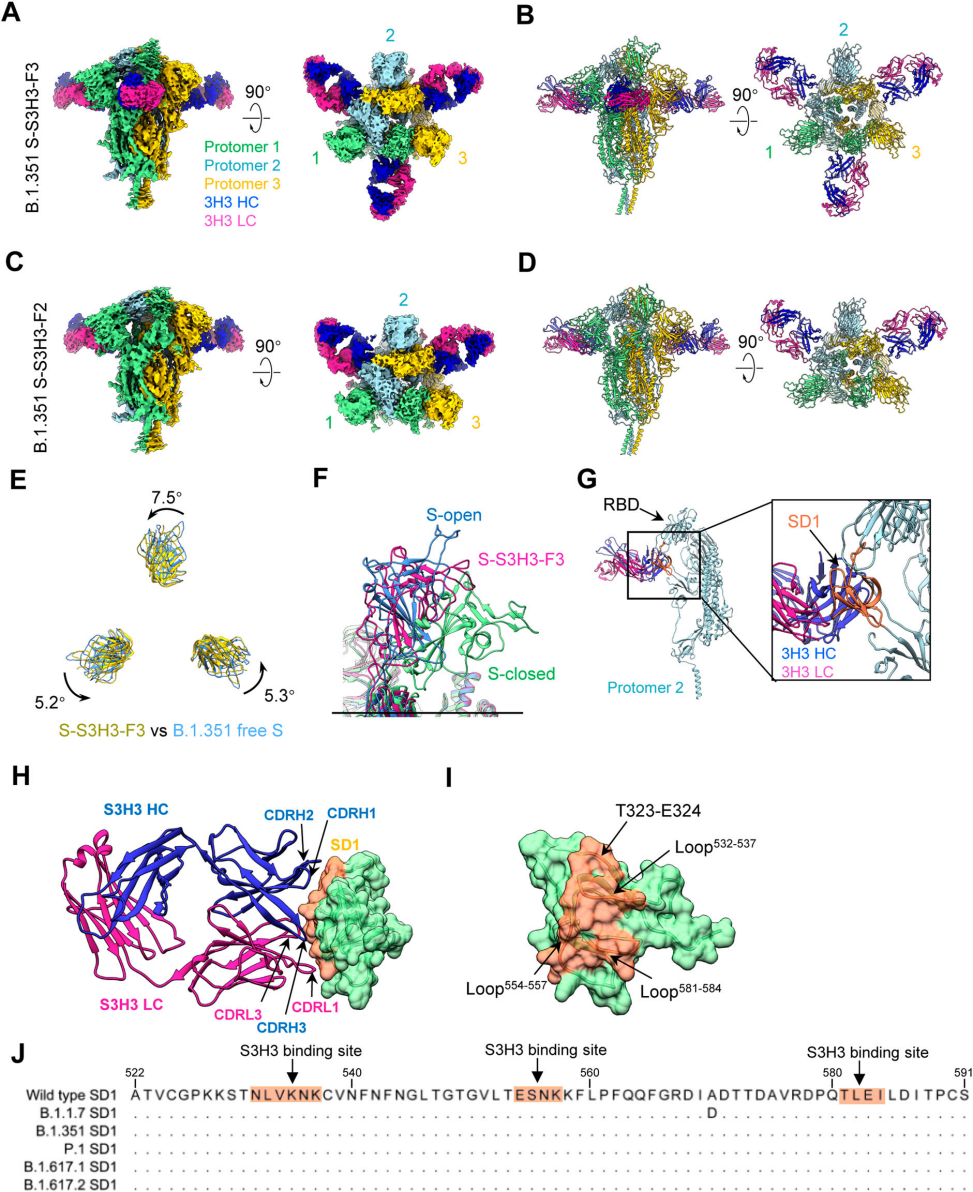
Cryo-EM structures of the SARS-CoV-2 B.1.351 S trimer in complex with S3H3 Fab. (A and B) Side and top views of the B.1.351 S-S3H3-F3 cryo-EM map (A) and pseudo atomic model (B). Only RBD-1 is “up.” Heavy and light chain of S3H3 Fab are shown in medium blue and violet red, respectively. (C and D) Side and top views of the S-S3H3-F2 cryo-EM map (C) and pseudo atomic model (D). (E) Conformational comparation between B.1.351 S-S3H3-F3 and the open state of B.1.351 S trimer (PDB: 7VX1). (F) RBD-1 of S-S3H3-F3 is in the transition state between “open” and “closed” (PDB: 7N1T) configuration. (G) S3H3 Fab binds SD1 of a protomer. (H and I) The interaction involved regions/residues between S3H3 Fab (H) and the SD1 (I). The S3H3 binding sites (coral) are indicated by arrows. (J) Sequence alignment of the SD1 region for different SARS-CoV-2 variants.

MAb S3H3, the only member of the group 3 antibody, broadly neutralizes the SARS-CoV-2 variants tested (Figure 2(C, D)). This antibody efficiently bound S trimer but did not react with RBD and NTD (Figure 1(D, E, F)). To reveal the structural basis for S3H3-mediated broad-spectrum neutralization, we resolved two cryo-EM maps of the SARS-CoV-2 B.1.351 S trimer in complex with S3H3 Fab in distinct conformational states, termed S-S3H3-F3 (associated with three Fab) and S-S3H3-F2 (with two Fabs), at 3.7 and 3.9 Å resolution, respectively (Figure 5(A–D); Supplementary Table S2; Supplementary Figure 10 and 11). The S-S3H3-F2/F3 maps show similar conformation with only one RBD in the “up” conformation and the remaining RBDs in the “down” conformation (Figure 5(A–D)). Compared with free B.1.351 S (PDB: 7VX1) [56], the S trimer in the S-S3H3-F3 structure exhibits a 5.2° to 7.5° twist (anti clockwise rotation) and tends to be less “open” as a whole (Figure 5(E)). Thus, the RBD-1 of S-S3H3-F3 appears in a transition state between “open” and “closed” (Figure 5(F)). This S3H3 Fab engagement-induced conformational change may lock the fusion machinery in the more prefusion state, hindering the shedding of S1 and the subsequent transformation towards the postfusion state.

Our structures showed that the S3H3 Fab is bound on the subdomain1 (SD1) region of S trimer (Figure 5(G)). We further focused refined the SD1-S3H3 Fab region and obtained a better resolved SD1-S3H3 map, which allowed us to examine the interaction interface between S3H3 Fab and B.1.351 S (Supplementary Figure 10; and Supplementary Figure 11(C, D)). Our structural analysis suggested that the heavy chain of S3H3 Fab contributes more to the interactions with SD1 than the light chain does, i.e. all the three heavy-chain CDRs of S3H3 and its CDRL1 and CDRL3 interact with the SD1 (Figure 5(H) and Supplementary Table S4). Specifically, the CDRs of S3H3 contact the T323-E324 and three loops (loop^532–537^, loop^554–557^ and loop^581–584^) of SD1, constituting an interaction network that facilitates the tightly binding between S3H3 Fab and SD1 (Figure 5(I)). The SD1 region targeted by S3H3 is an evolutionarily stable region among SARS-CoV-2 strains (Figure 5(J)), whereas it differs significantly from those of other human betacoronaviruses (Supplementary Figure 12). Mutations occurring in the SARS-CoV-2 B.1.1.7, B.1.351, B.1.617.1, and B.1.617.2 variant S proteins relative to the WT S are not present in the S3H3-SD1 interaction interface. Thus, these mutations will not affect S3H3 binding, explaining the observed broad-spectrum SARS-CoV-2 neutralization potency of S3H3 Mab.

## Discussion

In this study, we comprehensively characterized 9 neutralizing MAbs raised against the S protein of the SARS-CoV-2 ancestral strain. These MAbs can be divided into three groups based on their binding target: the group 1 comprises four RBD-binding MAbs; the group 2 contains four NTD-binding MAbs; and the group 3 has a single member that reacts with neither RBD nor NTD. Some MAbs in the groups 1 and 3 exhibited cross-neutralization towards a panel of variant pseudoviruses. By using a combination of biochemical, virological and structural approaches, we defined the binding epitopes of these broadly neutralizing antibodies, one of which has not been reported previously.

The MAbs in the group 1 showed diverse neutralization patterns towards the pseudovirus panel (Figure 2(C)). Specifically, S1D8 and S5D2 exhibited comparable neutralization against the B.1.1.7, B.1.351, and B.1.617.1 variants relative to the original strain, but showed drastically reduced potency (by at least 45 folds) towards the B.1.617.2; the neutralizing potency of MAb S4G8 decreased significantly for each of the variants tested; whereas S5G2 could neutralize all of the four variants with similar potency. Epitope mapping showed that S1D8 and S5D2 bind RBM whereas S5G2 targeted primarily the core region and occupied the binding site of 3C1, a previously identified core-binding MAb [37] (Figure 3). These results define two types of RBD-directed broadly neutralizing antibodies: type I, exemplified by S5D2, binds the RBM and has a medium-range cross-neutralization capacity (except B.1.617.2); and type II, represented by S5G2, binds the RBD core and displays a broad neutralization spectrum against the major emerged variants. Both S5D2 and S5G2 can block ACE2 binding to WT S trimer, suggesting that ACE2 blockade is the main neutralization mechanism for these two types of broadly neutralizing MAbs. Based on an early classification scheme for RBD antibodies [16,57], S5D2 and S5G2 can be assigned to the antibody classes I and III, respectively. Hastie et al., has recently proposed a new grouping scheme which categorizes RBD antibodies into seven major communities (RBD-1 to RBD-7) [58]. According to this updated scheme, S5D2 and S5G2 belong to the RBD-2 and RBD-7 antibody communities, respectively.

Our structural studies revealed that S5D2 binds at the top lateral edge of the RBM with a binding footprint centred around the loop^477–489^. The mutations occurred on the RBDs of B.1.1.7 (N501Y), B.1.351 (K417N, E484 K, and N501Y), and B.1.617.1 (L452R and E484Q) are not the contact residues of S5D2 Fab, thus explaining the observed neutralization potency of S5D2 against these variants. However, the T478 K mutation site on the B.1.617.2 RBD is located within the binding epitope of S5D2 Fab. The surface property alternation and side chain enlargement induced by the T478 K replacement in B.1.617.2 may break the interaction network with S5D2 Fab, leading to reduced S5D2 binding and increased resistance to neutralization by S5D2. The binding mode of S5D2 is very similar to those of previously reported human MAbs, S2E12 [29,59] and A23-58.1 [30]. Notably, the same mutation site that confers antibody resistance was positively selected in vitro in the presence of S5D2 (F486L) or A23-58.1 (F486S) [30], indicating that S5D2 and A23-58.1 share the same contact residue F486 on the RBD. Consistent with the neutralization profile of our S5D2 MAb, both S2E12 and A23-58.1 also retained high neutralizing potency against B.1.1.7 and B.1.351 [29,30,59], while their potency against B.1.617.2 has not been reported yet. It should be mentioned that the F486L mutation selected by S5D2 in vitro is also detected in natural SARS-CoV-2 strains with very low frequency (less than 1/12,000) [60,61], suggesting that this mutation may not have significant impact on viral fitness or transmissibility.

S5G2 is a RBD core-targeting antibody with broad neutralization spectrum. In the antibody competition assay (Figure 3(D)), S5G2 almost completely abolished the RBD binding by the previously characterized antibody 3C1 [37], suggesting that the two MAbs may share the same epitope. As our previous work has structurally defined the binding epitope of 3C1, which involves mainly the β2-strand (T376 to C379) and loop^380–385^ [37], to minimize unnecessary work we elected not to determine the structure of S5G2 Fab in complex with S trimer in this study. Nonetheless, we found, in the escape mutant selection experiment, that K378 is a key residue determining S5G2’s binding and neutralization, thus verifying the epitope (Figure 3(G, H)). The S5G2/3C1 epitope is highly conserved among SARS-CoV-2 variants and even between SARS-CoV-2 and SARS-CoV (Supplementary Figure 5), thus explaining the observed cross-variant neutralization by S5G2. Also, S5G2 was found to efficiently neutralize the SARS-CoV pseudovirus (Supplementary Figure 5). Our data suggest that RBD core-targeting MAbs like S5G2, as broad-spectrum neutralizers, should be considered for inclusion into therapeutic antibody cocktail.

In this study, we identified 4 NTD-targeting MAbs that neutralize the original SARS-CoV-2 strain. However, they are not broad-spectrum neutralizers as indicated by significantly decreased potency against at least two out of the four variants tested, especially B.1.351 and B.1.617.2 (Figure 2(A)). Previous reports have also shown that B.1.351 is refractory to neutralization by most, if not all, of the NTD MAbs isolated during early phase of the COVID19 pandemic, especially those targeting the supersite [19,29,31]. Compared with RBD-directed MAbs which have a disperse epitope distribution [19], antibodies to NTD share a relatively single epitope, the so-called “supersite”, shaped by the N1 loop, N3 loop and N5 loop [19,23], thus creating a concentrated region under immune selective pressure. In addition, as the NTD “supersite” is not directly involved in the interaction between SARS-CoV-2 and ACE2 receptor, mutations within this site are therefore likely be well tolerated by the virus. In fact, mutations such as L18F, DEL144, DEL242-244, and R246I, which are detected in multiple SARS-CoV-2 variants, are located within or near the N1 loop, N3 loop and N5 loop [23]. As the consequence, NTD antibodies showed significantly reduced or even completely abolished neutralization activities towards major SARS-CoV-2 variants [31]. Collectively, these findings suggest that NTD may not harbour broadly neutralizing sites.

Perhaps the most significant finding in this study is the discovery of a SD1-targeting neutralizing MAb, S3H3, representing a previously unreported class of SARS-CoV-2 neutralizing antibodies. S3H3 exhibits comparable neutralization towards the four variants tested. Consistently, our structural study shows that the S3H3 epitope involves three loops (loop^532–537^, loop^554–557^ and loop^581–584^) of SD1 and possibly two residues (T323 and E324) of RBD, all of which are identical among the major SARS-CoV-2 variants (Figure 5(J) and Supplementary Figure 5). Indeed, the complete SD1 region (A522 to S591) is nearly identical among the major variants (except B.1.1.7 contains an A570D mutation which is buried in S trimer and hence will not contribute to antibody recognition). Hence, the S3H3 epitope represents the third (type III), yet previously unreported, broadly neutralizing site defined in this study. S3H3 does not block ACE2 binding to S trimer (Supplementary Figure 3D). Our structural analysis showed that S3H3 binding tightens the B.1.351 S trimer with its RBD-1 in a transition state between “open” and “closed” configuration, inferring that S3H3 may inhibit viral entry by locking the fusion machinery in the prefusion state and/or by affecting RBM exposure to cellular ACE2 receptor. Nonetheless, the exact mechanism of S3H3-mediated neutralization remains to be elucidated by further experimentation.

Several recent studies have identified neutralizing MAbs that bind the S2 region of the SARS-CoV-2 spike protein [25,62–64]. These S2-targeting antibodies were in general much less potent in neutralizing SARS-CoV-2 than RBD- or NTD-directed antibodies, however they exhibited broader neutralization spectrums. For example, the antibody S2P6 that binds the stem helix in the S2 region could neutralize SARS-CoV-2 variants (including Alpha, Beta, Gamma, and Kappa) and also cross-neutralize other betacoronaviruses such as SARS-CoV and MERS-CoV, albeit with relatively low efficiency [64], thus demonstrating that the S2 region does harbour broadly neutralizing epitopes. We should mention that, in the present study, we did not obtain S2-directed neutralizing MAbs. Although we used S-trimer as the immunogen which theoretically could induce S2-targeting antibodies besides RBD and NTD antibodies, we performed pseudovirus neutralization assay to screen hybridoma clones and only selected high- and medium-potency neutralizing antibodies for subsequent in-depth analyses. It is thus possible that S2 antibody clones with low neutralization efficiency could have been missed in our initial screening.

In summary, the present study identifies three types of SARS-CoV-2 cross-variant neutralizing MAbs, including a previously unreported one that targets the highly conserved SD1 region of the S protein, allowing us to define three distinct broadly neutralizing sites. These MAbs and their epitope information may be valuable for design and development of broadly effective vaccines and MAb-based therapies.

## Supplementary Material

Supplemental Material

## Data Availability

All data needed to evaluate the conclusions in the paper have been present in the paper and/or the Supplementary Materials. For the S-S5D2 Fab dataset, related cryo-EM maps have been deposited in the Protein Data Bank with accession codes 7WCZ, 7WD0, 7WD7, and 7WCR, and the associated models have been deposited in the Electron Microscopy Data Bank with accession codes EMD-32430, EMD-32431, EMD-32433, and EMD-32428 for S-S5D2-F1, S-S5D2-F2, S-S5D2-F3, and RBD-1-S5D2, respectively. For the S-S3H3 Fab dataset, related cryo-EM maps have been deposited in the Protein Data Bank Bank with accession codes 7WD9, 7WDF, and 7WD8, and the associated models have been deposited in the Electron Microscopy Data Bank with accession codes EMD-32435, EMD-32437 and EMD-32434 for S-S3H3-F3, S-S3H3-F2, and SD1-3H3, respectively.
