# Supplementary material for: Mapping cross-variant neutralizing sites on the SARS-CoV-2 spike protein
Source: Emerg Microbes Infect. 2022 Jan 24;11(1):351–67. doi: 10.1080/22221751.2021.2024455 (PMC8794075; doi:10.1080/22221751.2021.2024455)
Supplement: Supplemental Material [file TEMI_A_2024455_SM2890.docx]

**Table S1. Neutralization ability of hybridoma culture supernatants against wild-type (WT) SARS-CoV-2 or SARS-CoV pseudoviruses.** For each hybridoma clone, 45μl of culture supernatant was mixed with the indicated pseudovirus and tested for neutralization. Results were expressed as follows: “+”, > 85% neutralization; “–”, < 15% neutralization.

| Hybridoma  clone | Antibody  isotype | Neutralization of pseudovirus | |
| --- | --- | --- | --- |
|  |  | WT SARS-CoV-2 | WT SARS-CoV |
| S1D8 | IgG1 | **+** | **-** |
| S2G4 | IgG1 | **+** | **-** |
| S2H5 | IgG1 | **+** | **-** |
| S3H3 | IgG1 | **+** | **-** |
| S4D4 | IgG1 | **+** | **-** |
| S4G8 | IgG1 | **+** | **-** |
| S5B8 | IgG1 | **+** | **-** |
| S5D2 | IgG1 | **+** | **-** |
| S5G2 | IgG2b | **+** | **+** |

**Table S2. Cryo-EM data collection and refinement statistics for B.1.351 S-S5D2 and B.1.351 S-S3H3 complexes**

|  | B.1.351 S-S5D2 | | | | B.1.351 S-S3H3 | | | | | |  |
| --- | --- | --- | --- | --- | --- | --- | --- | --- | --- | --- | --- |
| **Data collection** |  | | | | | | | | | |  |
| EM equipment | Titan Krios | | | | Titan Krios | | | | | |  |
| Voltage (kV) | 300 | | | | 300 | | | | | |  |
| Detector | K3 | | | | K3 | | | | | |  |
| Pixel size (Å) | 1.093 | | | | 1.093 | | | | | |  |
| Electron dose (e^-^/Å^2^) | 50 | | | | 50 | | | | | |  |
| Exposure time (s) | 3 | | | | 3 | | | | | |  |
| Frames | 30 | | | | 30 | | | | | |  |
| Defocus range (μm) | -0.8 to -2.5 | | | | -0.8 to -2.5 | | | | | |  |
| **Reconstruction** |  | | | | | | | | | |  |
| Softwares | Relion 3.1& cryoSPARC | | | | | | | | |  |  |
| Structures | S5D2-F1 | S5D2-F2 | S5D2-F3 | RBD-1-S5D2 | | S3H3-F3 | S3H3-F2 | | SD1-S3H3 | | |
| Final particles | 35,322 | 129,238 | 35,225 | 100,064 | | 108,300 | 53,200 | | 102,262 | | |
| Symmetry | C1 | C1 | C1 | C1 | | C1 | C1 | | C1 | | |
| Final resolution (Å) | 3.5 Å | 3.3 Å | 3.5 Å | 3.5 Å | | 3.7 Å | 3.9 Å | | 4.3 Å | | |
| **Atomic modeling** |  | | | | | | | | | |  |
| Softwares | Rosetta & Phenix & Coot | | | | | | | | | |  |
| Rms deviations | | | | | | | | | | |  |
| Bond length (Å) | 0.0090 | 0.0041 | 0.0034 | 0.0040 | | 0.0037 | 0.0035 | | 0.0033 | | |
| Bond Angle (°) | 1.04 | 1.04 | 0.96 | 1.02 | | 0.92 | 0.92 | | 0.94 | | |
| Ramachandran plot (%) | | | | | | | | | | |  |
| Favored | 96.38 | 96.32 | 95.35 | 96.95 | | 95.64 | 95.60 | 95.74 | | | |
| Allowed | 3.62 | 3.68 | 4.54 | 2.73 | | 4.25 | 4.28 | 4.07 | | | |
| Outliers | 0.00 | 0.00 | 0.11 | 0.32 | | 0.11 | 0.12 | 0.19 | | | |
| Molprobity score | 1.45 | 1.33 | 1.75 | 1.52 | | 1.35 | 1.71 | 1.77 | | | |
| Clash score | 4.35 | 2.91 | 7.72 | 4.39 | | 2.58 | 7.38 | 8.80 | | | |

**Table S3. Contacting residues (with a side chain distance cut off 4 Å) at the SARS-CoV-2 B.1.351 RBD/S5D2 interface.**

| B.1.351 S RBD-1 | S5D2 |
| --- | --- |
| F456* | N54 |
| S477 | Y97, A102 |
| T478 | Y97, Y100 |
| P479 | Y31, Y97, Y98 |
| C480 | Y31 |
| N481 | Y31 |
| F486* | Y100, Y35, N52, D57, T59 |
| N487* | T33 |
| Y489* | N52, D55 |

Heavy chain

Light chain

* ACE2 binding sites

**Table S4.** Contacting residues (with a side chain distance cut off 4 Å) at the SARS-CoV-2 B.1.351 S/3H3 interface.

| B.1.351 S RBD-1 | S3H3 |
| --- | --- |
| T323 | H52, S54 |
| E324 | R31 |
| N532 | F32 |
| L533 | Y101 |
| V534 | R31, W33, Y101 |
| K535 | W33, Y101, Y103 |
| N536 | L98, W33 |
| K537 | H52, D55, E57 |
| E554 | Y36, S95, R96 |
| S555 | A31, S32 |
| N556 | A31 |
| K557 | S32 |
| T581 | D102 |
| L582 | Y34 |
| E583 | Y103 |
| I584 | S32 |

Heavy chain

Light chain


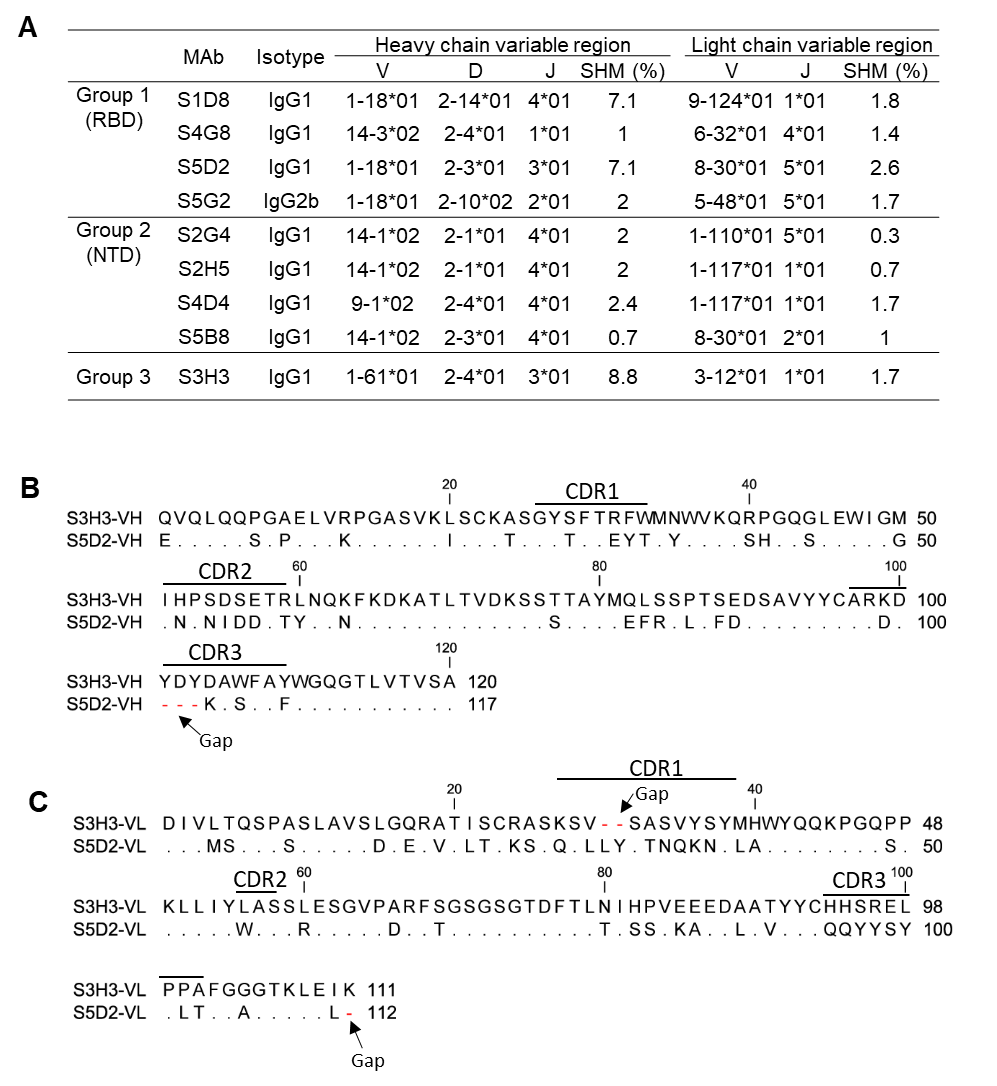


**Figure S1. Sequence analysis of the variable regions of the SARS-CoV-2 MAbs.**

(**A**) The closest-matched germline V, D and J genes identified by IgBLAST. SHM, somatic hypermutation. (**B-C**) Heavy (**B**) and light (**C**) chain variable region (VH and VL) amino acid sequences of MAbs S3H3 and S5D2. Dots represent residues identical to those of MAb S3H3, and red dashes are gaps. Locations of complementarity determining regions (CDR) were indicated.


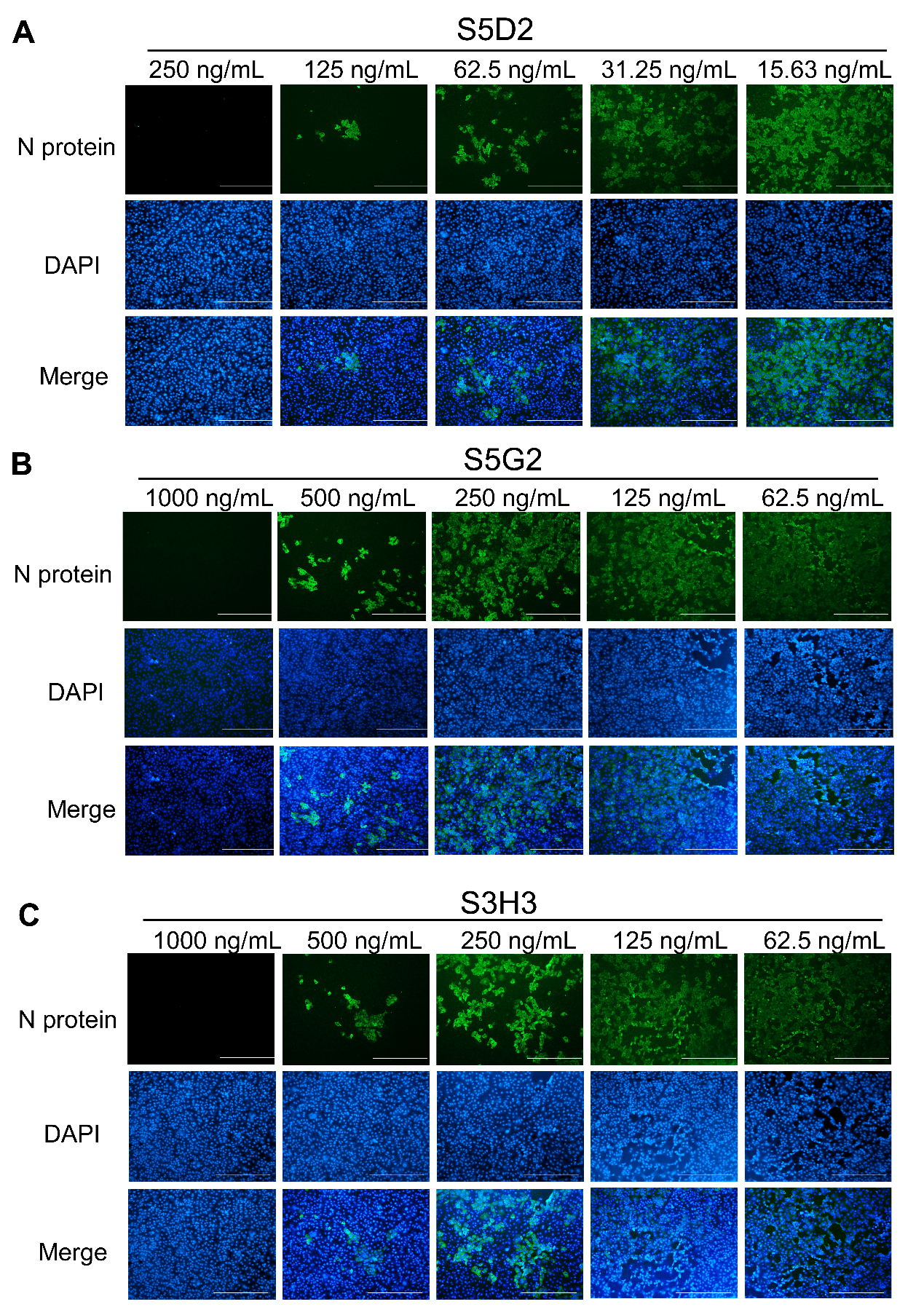


**Figure S2. Neutralization of authentic wild type SARS-CoV-2 by MAbs.** Live SARS-CoV-2 virus was incubated with serially diluted MAb prior to addition to VeroE6 cells. After two days, the cells were fixed and stained with N protein-specific antibody. Representative immunofluorescence images for MAbs S5D2 **(A)**, S5G2 **(B)**, and S3H3 **(C)** are shown. Bar, 400 μm.


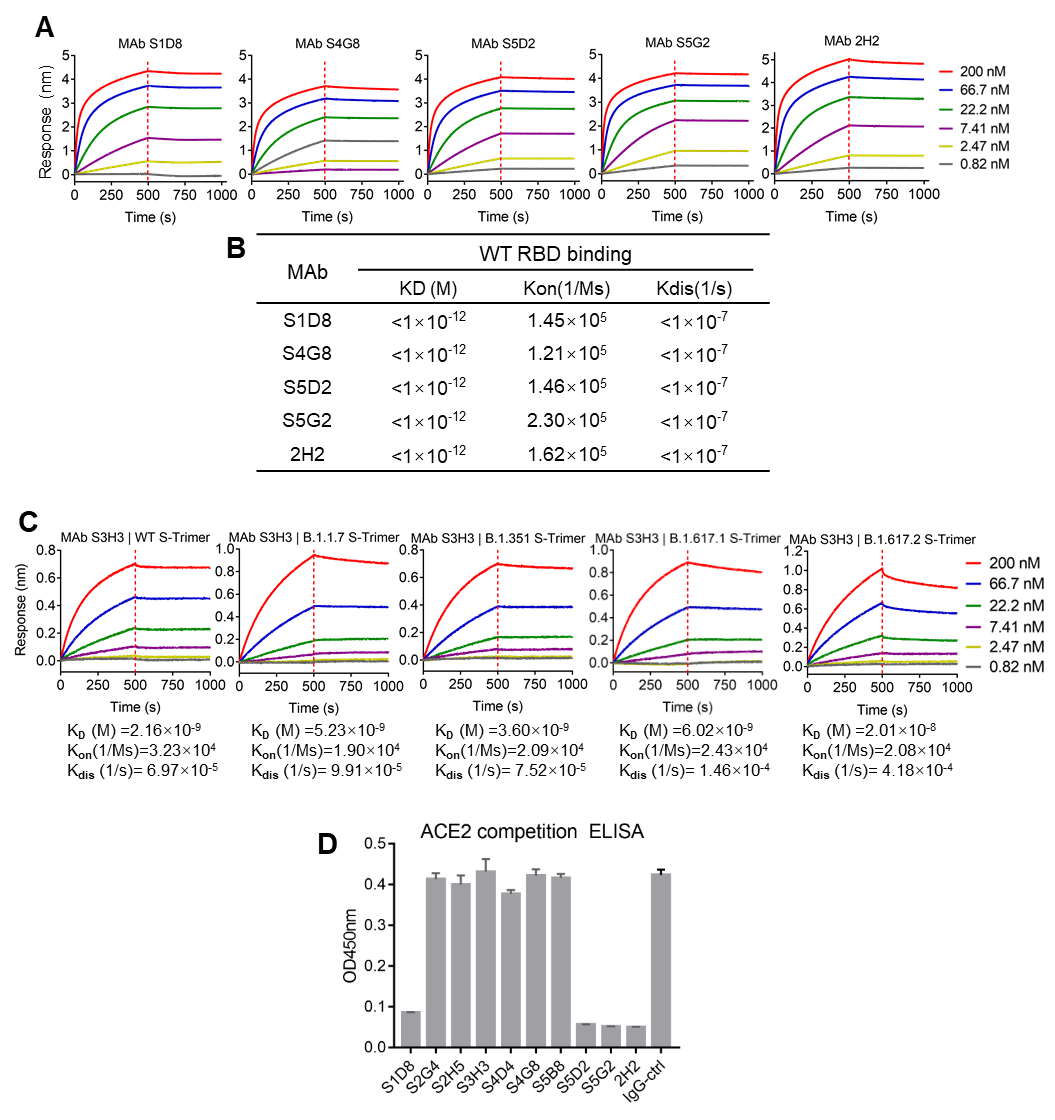


**Figure S3. Biochemical characterization of MAb binding. (A)** Binding kinetics of the RBD-reactive MAbs to wild-type (WT) SARS-CoV-2 RBD measured by BLI. Association and dissociation steps are divided by dotted red line. Antibody concentrations tested are shown. **(B)** Summary of the binding affinity of the MAbs to WT RBD. **(C)** Binding affinity of S3H3 to S trimers from SARS-CoV-2 WT and variants measured by BLI. Antibody concentrations tested are shown. **(D)** Competition between the MAbs and ACE2 for binding to WT RBD was determined by ELISA. The ACE2-binding signal was detected by a corresponding secondary antibody. Data are mean ± SD of triplicate wells.


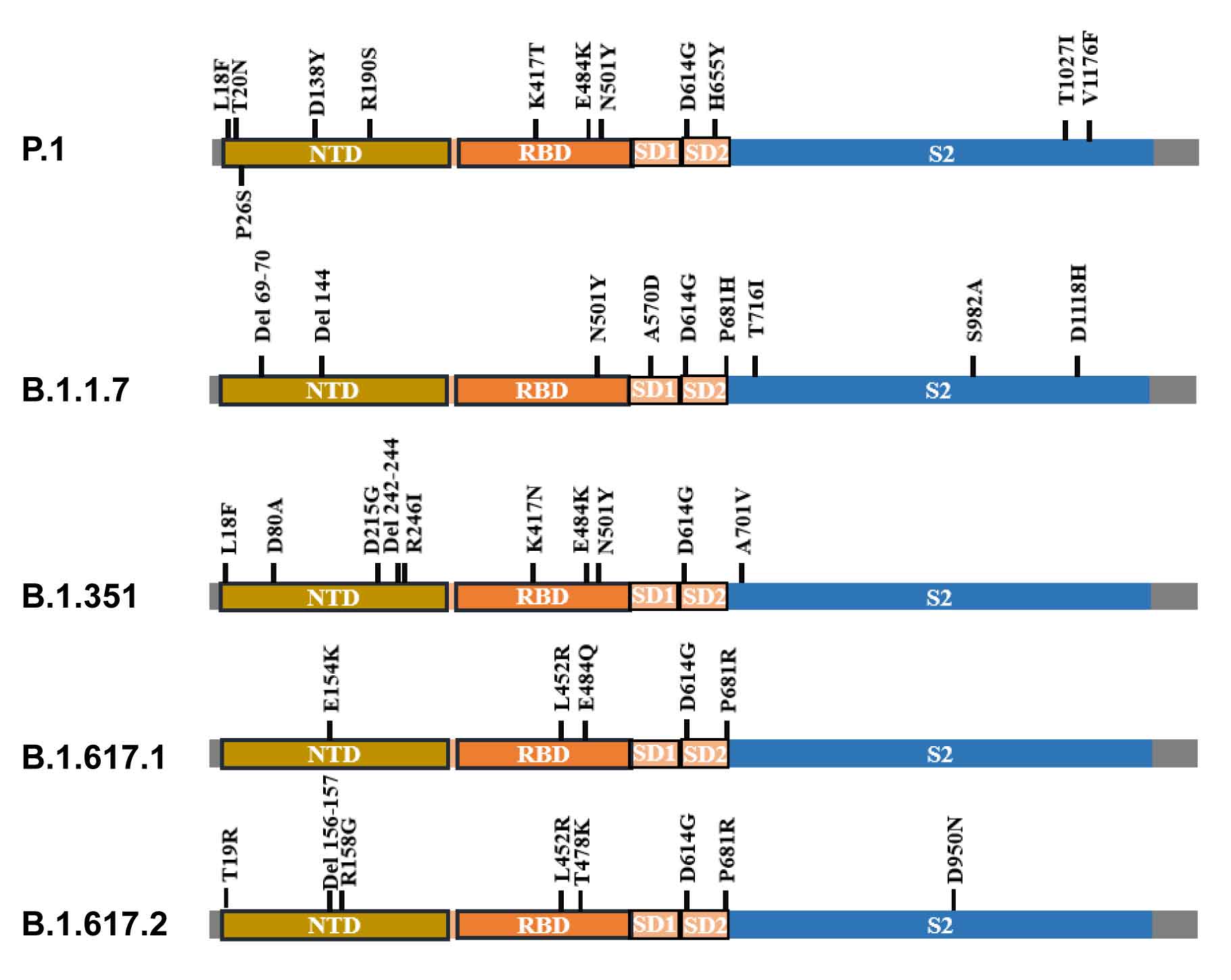


**Supplementary Figure 4.** Schematic diagrams of the spikes of SARS-CoV-2 variants. Mutations in the P.1, B.1.1.7, B.1.351, B.1.617.1 and B.1.617.2 variants are shown at the top of each diagram.


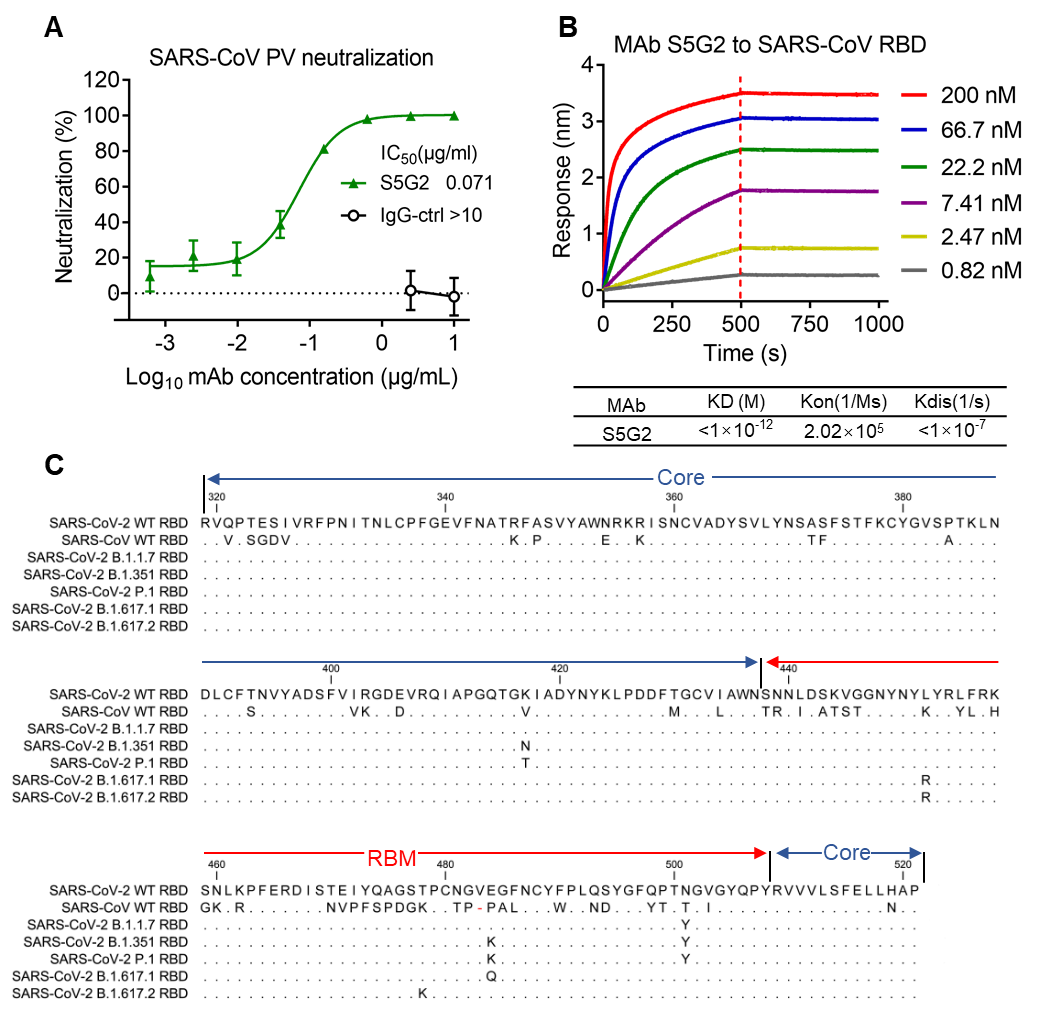


**Figure S5. Neutralization activity and binding affinity of MAb S5G2 against SARS-CoV. (A)** Neutralization activity of MAb S5G2 against SARS-CoV pseudovirus. Data are expressed as mean ± SD of four replicate wells. **(B)** Binding affinity of MAb S5G2 to immobilized SARS-CoV RBD measured by BLI. Association and dissociation steps are divided by dotted red line. S5G2 concentrations used are shown. **(C)** Amino acid sequence alignment for the RBD region among SARS-CoV-2 WT, B.1.1.7, B.1.351, P.1, B.1.617.1, B.1.617.2 strains and SARS-CoV. The Core and RBM regions in the RBD are indicated.


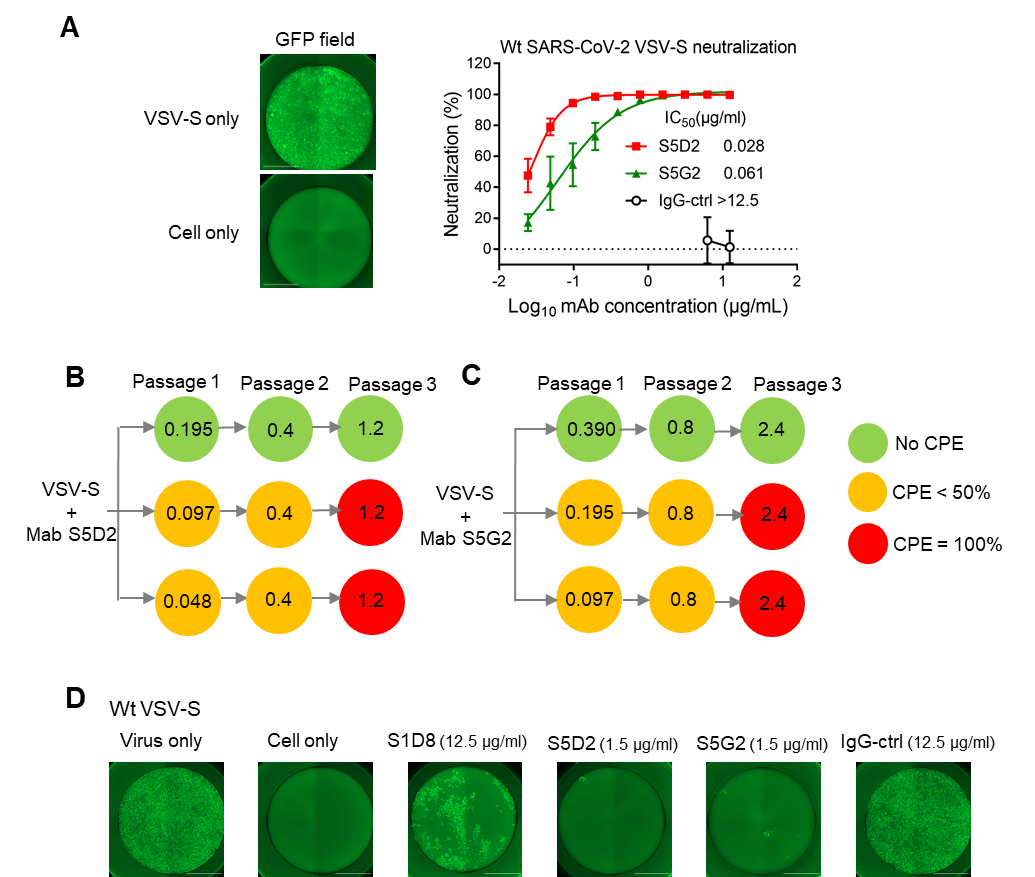


**Figure S6. Neutralization activity of the MAbs against VSV-SARS-CoV-2-S (VSV-S) and selection of neutralization-resistant mutants. (A)** Neutralization activity of MAbs S5D2 and S5G2 against VSV pseudotyped with SARS-CoV-2 S protein (VSV-S). Data are mean ± SD of four replicate wells. The infection of VSV-S, which contained a GFP reporter gene, produced fluorescent signals (left panel). **(B-C)** Selection of neutralization-resistant mutants. VSV-S was passaged under increasing concentrations of MAb S5D2 **(B)** or S5G2 **(C)**, and the cells were observed for cytopathic effects (CPE). MAb concentrations used in each passage are shown in the circles. Green circle indicates no CPE, orange circle indicates that CPE was observed in < 50% of the cells, and red circle indicates complete CPE. **(D)** Neutralization activity of MAbs S5D2, and S5G2 against parental VSV-S. Antibody concentrations used were shown in parentheses.


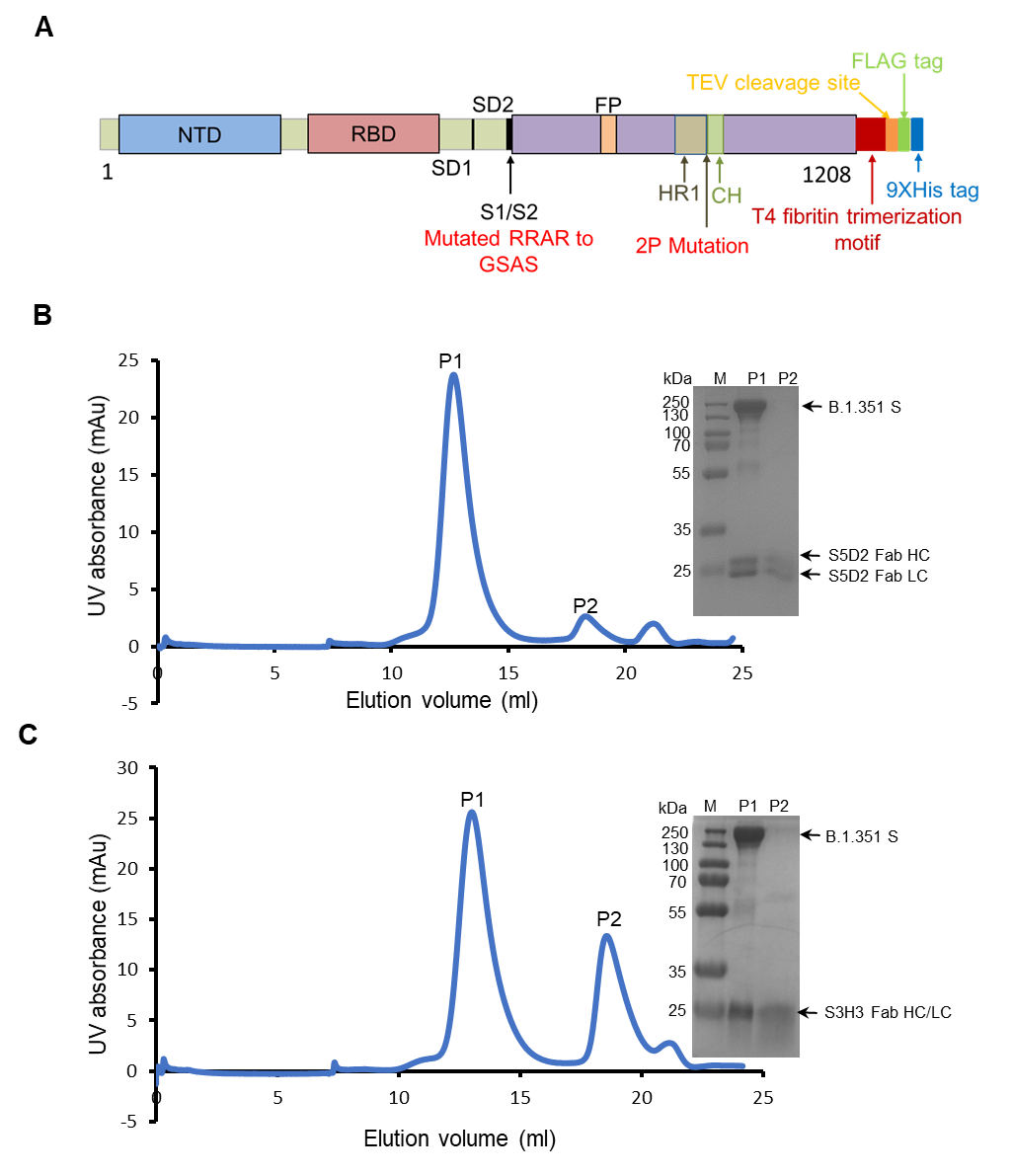


**Figure S7. (A)** Schematic diagram of S-trimer. **(B-C)** Size-exclusion chromatogram and SDS-PAGE analysis of the S5D2/B.1.351 S-trimer complex **(B)** and S3H3/B.1.351 S-trimer complex **(C)**.


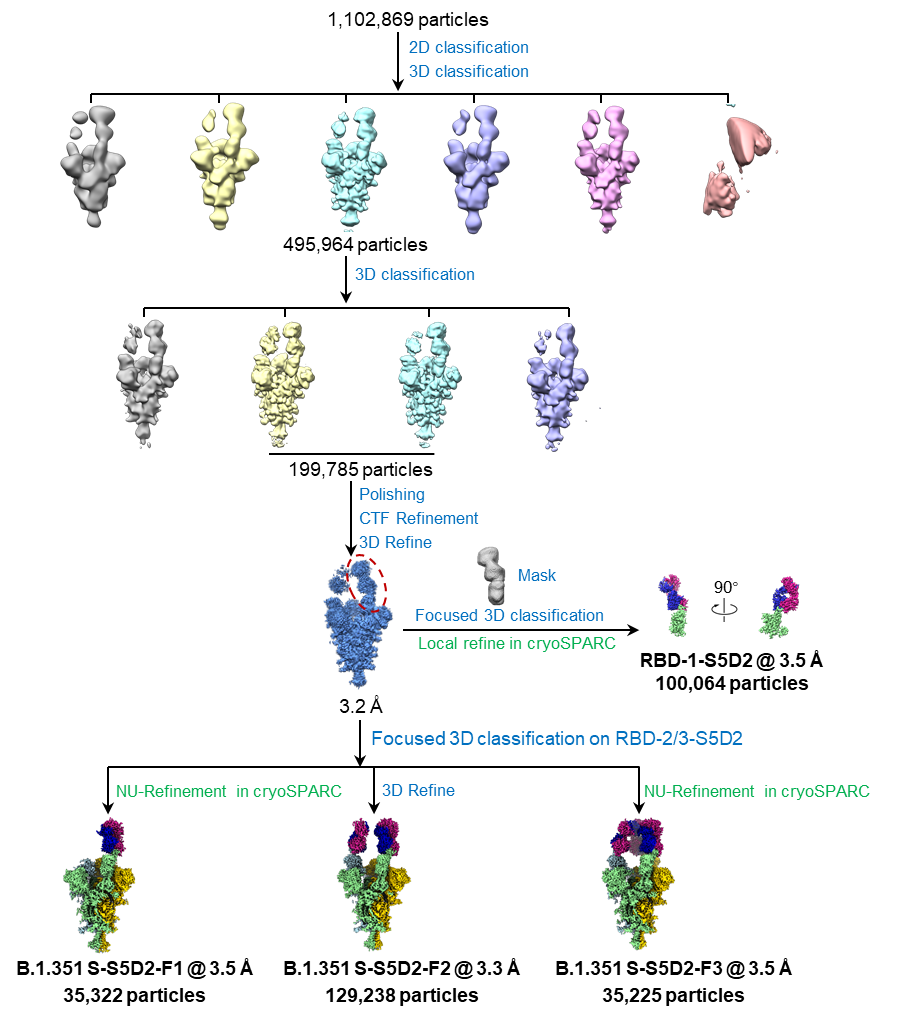


**Figure S8. Cryo-EM data processing procedure for SARS-CoV-2 B.1.351 S-S5D2 dataset.**


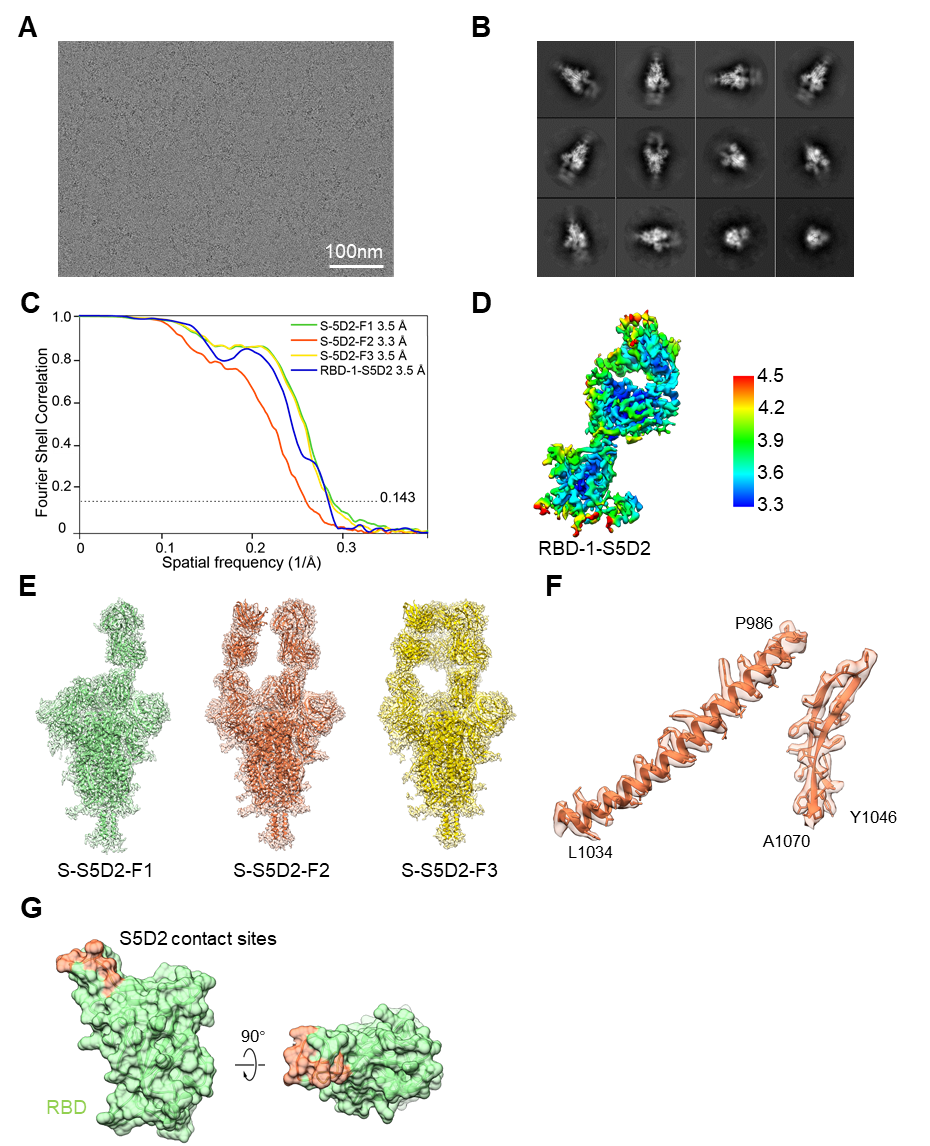


**Figure S9. Cryo-EM analysis on the SARS-CoV-2 B.1.351 S-S5D2 complex. (A)** Representative cryo-EM image of the S trimer in the presence of S5D2 Fab. **(B)** Reference-free 2D class averages of the S-S5D2 complex. **(C)** Resolution assessment of the cryo-EM reconstructions by Fourier shell correlation (FSC) at 0.143 criterion. **(D)** Local resolution of RBD-1-S5D2 map. **(E)** Model-map fitting of S-S5D2-F1, S-S5D2-F2, and S-S5D2-F3 structure. **(F)** Representative high-resolution structural features of the S-S5D2-F2 map. **(G)** S5D2 contact sites (coral) on RBD.


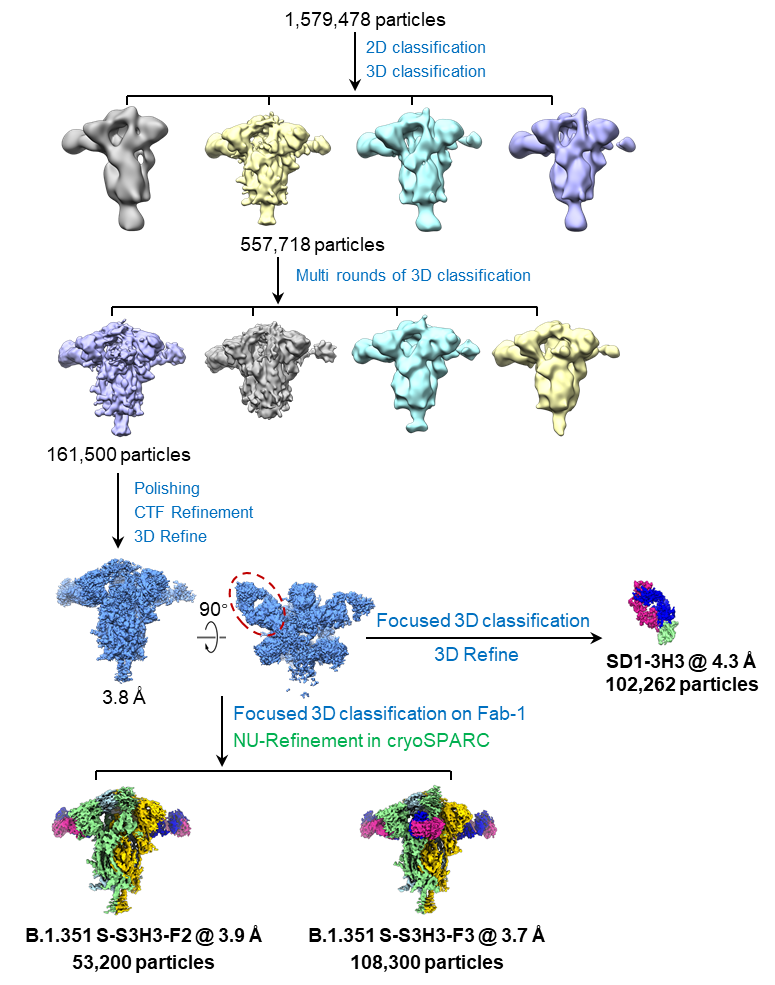


**Figure S10 Cryo-EM data processing procedure for SARS-CoV-2 B.1.351 S-S3H3 dataset.**


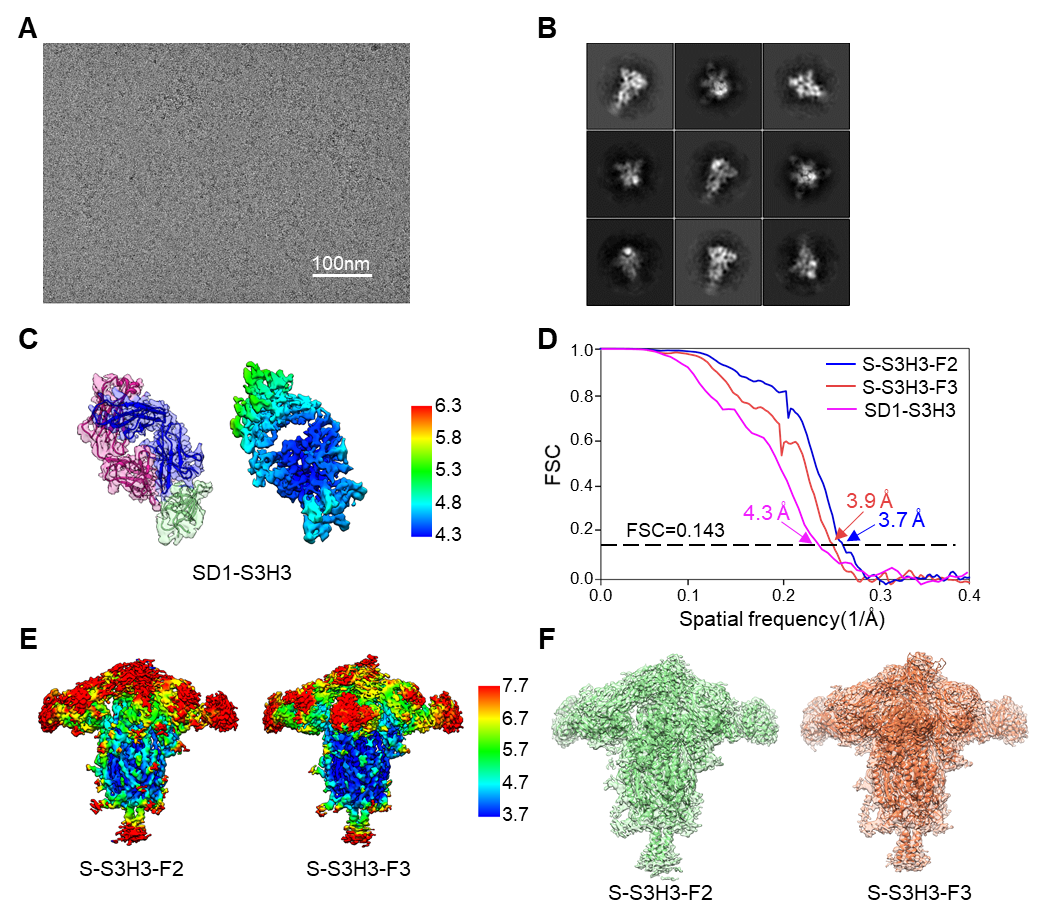


**Figure S11. Cryo-EM analysis on the SARS-CoV-2 B.1.351 S-S3H3 complex. (A)** Representative cryo-EM image of the S trimer in the presence of S3H3 Fab. **(B)** Reference-free 2D class averages of the S-S3H3 complex. **(C)** Focus-refined cryo-EM map and local resolution of SD1-S3H3 Fab. **(D)** Resolution assessment of the cryo-EM reconstructions by Fourier shell correlation (FSC) at 0.143 criterion. **(E)** Local resolution of the S-S3H3-F2 and S-S3H3-F3 maps. **(F)** Model-map fitting of the S-S3H3 complex.


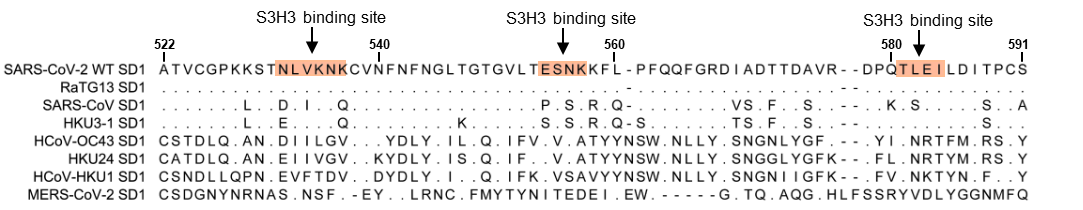


**Figure S12. Amino acid sequence alignment of the corresponding SD1 regions from different human betacoronaviruses.**
